# Timing matters: age-dependent impacts of the social environment and host selection on the avian gut microbiota

**DOI:** 10.1186/s40168-022-01401-0

**Published:** 2022-11-26

**Authors:** Öncü Maraci, Anna Antonatou-Papaioannou, Sebastian Jünemann, Kathrin Engel, Omar Castillo-Gutiérrez, Tobias Busche, Jörn Kalinowski, Barbara A. Caspers

**Affiliations:** 1 Department of Behavioural Ecology, Bielefeld University, Bielefeld, Germany; 2 Evolutionary Biology, Bielefeld University, Bielefeld, Germany; 3 Institute of Biology-Zoology, Freie Universität Berlin, Berlin, Germany; 4 Institute for Bio- and Geosciences, Research Center Jülich, Jülich, Germany; 5 Faculty of Technology, Bielefeld University, Bielefeld, Germany; 6 Center for Biotechnology (CeBiTec), Bielefeld University, Bielefeld, Germany

**Keywords:** Avian gut microbiome, Establishment of the gut microbiota, Social transmission, Host selection, Early development, Bengalese finch, Zebra finch, Succession, Ontogenesis

## Abstract

**Background:**

The establishment of the gut microbiota in early life is a critical process that influences the development and fitness of vertebrates. However, the relative influence of transmission from the early social environment and host selection throughout host ontogeny remains understudied, particularly in avian species. We conducted conspecific and heterospecific cross-fostering experiments in zebra finches (*Taeniopygia guttata*) and Bengalese finches (*Lonchura striata domestica*) under controlled conditions and repeatedly sampled the faecal microbiota of these birds over the first 3 months of life. We thus documented the development of the gut microbiota and characterised the relative impacts of the early social environment and host selection due to species-specific characteristics and individual genetic backgrounds across ontogeny by using 16S ribosomal RNA gene sequencing.

**Results:**

The taxonomic composition and community structure of the gut microbiota changed across ontogenetic stages; juvenile zebra finches exhibited higher alpha diversity than adults at the post-breeding stage. Furthermore, in early development, the microbial communities of juveniles raised by conspecific and heterospecific foster parents resembled those of their foster family, emphasising the importance of the social environment. In later stages, the social environment continued to influence the gut microbiota, but host selection increased in importance.

**Conclusions:**

We provided a baseline description of the developmental succession of gut microbiota in zebra finches and Bengalese finches, which is a necessary first step for understanding the impact of the early gut microbiota on host fitness. Furthermore, for the first time in avian species, we showed that the relative strengths of the two forces that shape the establishment and maintenance of the gut microbiota (i.e. host selection and dispersal from the social environment) change during development, with host selection increasing in importance. This finding should be considered when experimentally manipulating the early-life gut microbiota. Our findings also provide new insights into the mechanisms of host selection.

Video Abstract

**Supplementary Information:**

The online version contains supplementary material available at 10.1186/s40168-022-01401-0.

## Introduction

Microbial communities that inhabit the gastrointestinal tract of animals (collectively known as the gut microbiota) have metabolic functions that complement host physiology and influence numerous phenotypic traits of their hosts [1]. Initial microbial colonisers are deterministic in the establishment of long-term symbiotic interactions in animals [2]. They also play essential roles in host development, such as priming the immune system [3, 4] and facilitating the development of the nervous system [5, 6]. Dysbiosis of these communities in early life is a hallmark of wide-ranging physiological, behavioural and developmental disorders [7–10]. Despite increasing awareness of the importance of microbial communities acquired during early life to the host’s overall fitness [11], much remains unknown about the processes that control the establishment and ontogenesis of the gut microbiota.

One of the forces that shape the establishment and maintenance of these communities is the dispersal of microbes among hosts and from the physical surroundings [12]. In viviparous species, microorganisms are transferred from the mother to offspring, inter alia, during embryonic development [13, 14] and birth [15]. However, this direct association between mother and offspring during embryonic and perinatal periods is missing in oviparous species, such as birds. In these species, chicks are thought to obtain their first microorganisms from their parents, their nestmates and the environment only after hatching (but see [16, 17] for in ovo colonisation of the gut). In altricial species, regardless of whether they are oviparous or viviparous, the transmission of microorganisms between parents and offspring occurs via parental care, for example, while feeding offspring and during physical contact [18, 19]. Furthermore, microbes can be transmitted among members of the social group [20] and from the postnatal environment [21, 22].

Whether these initial colonisers will be incorporated into the host’s long-term symbiotic repertoire or eliminated from the microbial pool depends on host selection [23, 24]. The gut habitat deterministically sculpts its symbiotic profile by selecting microbial species with particular niches from the initial pool. Host selection, to a varying extent, is mediated by species-specific host characteristics, such as anatomical and physiological conditions of the gut [25, 26] and the immune system [1, 27–29]. Furthermore, the individual genetic background might play a role in the establishment process [30–34]. For example, germ-free individuals inoculated with the gut microbiota of individuals from another species develop microbial profiles that resemble their conventional communities [35, 36]. Moreover, when inoculated with identical microbial colonies, different genetic strains of mice raised in germ-free conditions exhibit markedly different microbial profiles, although inoculation with different microbial communities resulted in different microbial profiles in the same mice strains [30]. These findings indicate that parental factors, the environmental pool of microorganisms, interactions in the rearing environment and ecological and host-specific factors can affect the establishment and maintenance of gut microbiota, leading to marked interspecies [9, 37–39] and intraspecies variation [38, 40–42]. Nevertheless, a comprehensive understanding of the relative strength of all influential factors that shape the establishment and ontogenesis of gut microbiota, particularly of those that involve differential transmission dynamics and host selection based on species- and individual-specific traits, is lacking. This knowledge gap partly originates from the complexity of polymorphic genetic mechanisms that regulate the microbiota and the intertwined nature of maternal transmission and host genetics in viviparous species.

Birds are ideal study organisms to study the relative strengths of external and host factors in the establishment of microbial communities due to the lack of direct microbial transmission between mother and offspring during embryonic development. This allows manipulation of the microbial milieu before hatching. Some researchers have studied the relative impact of the rearing environment and host factors by leveraging brood parasitism [43–47], in which the eggs of a parasitic species are laid in the nest of another species [48]. Additionally, cross-fostering experiments have been conducted in natural populations [49, 50]. However, the findings of these studies are mixed. Some concluded that the impact of host factors outweighs environmental factors [43, 46, 47], while others showed that the rearing environment is the primary driver of the microbial establishment [49, 50]. These inconsistent findings could be due to several confounders in natural settings. Therefore, Chen et al. [22] conducted a cross-fostering experiment under controlled conditions to address this problem. By cross-fostering zebra finch (*Taeniopygia guttata*) eggs into Bengalese finch (*Lonchura striata domestica*) nests, they demonstrated that the gut microbiota of zebra finch juveniles was more similar to that of the parents that reared them during early development, i.e. the first 10 days after hatching. However, nothing is known about later developmental periods or whether and how the strength of different forces changes over ontogeny.

Data on how initial microbial colonies are recruited and how microbial diversity and composition change over host ontogeny are scarce and contradictory. Most of our current understanding of the relative influence of host factors and the rearing environment on the gut microbiota relies on studies conducted in natural environments, where many confounding factors are present. For example, the apparent impact of the nestling environment on the gut microbiota might originate from changes in the pool of initial colonisers due to parental influences, the environmental reservoir of pre-existing microorganisms, dietary alterations and other environmental conditions that affect both the environmental reservoir and the host. This impedes investigation of the impact of different transmission dynamics. Another limitation of these studies is that they provide insights only into the very early stages of life, particularly the prefledging period when the immune system has not yet fully matured [51, 52]. However, as the gut microbiota changes throughout the lifespan of avian hosts [53–55], it is reasonable to assume that host ontogenetic changes might affect the relative strengths of host selection and transmission. This hypothesis has only been tested by a handful of studies in aquatic systems, which demonstrated that the strength of host selection increases with host maturation in fish [2, 56–58] and shrimp [59]. Although understanding the role of host and environmental factors on the microbiota has become a central theme in avian microbial ecology (reviewed in [60]), to the best of our knowledge, no studies have specifically investigated the relative influence of social transmission and host selection across the distinct developmental stages of birds.

In the present study, we aimed to investigate the impacts of social transmission and host selection on the establishment of the gut microbiota at different ontogenetic stages under controlled dietary and environmental conditions. We conducted our study on two estrildid finches: the zebra finch, a well-studied model organism [61], and the Bengalese finch, which is known to indiscriminately raise chicks from other finch species [62, 63]. Our previous works showed that the skin and gut microbiota of these species differ and that individuals of both species exhibit unique, temporally stable microbial features under controlled conditions [38, 39]. To study the relative impacts of host genetics and environmental factors, we manipulated the prenatal environment by (i) cross-fostering eggs between different pairs of zebra finches, (ii) cross-fostering eggs between different pairs of Bengalese finches and (iii) cross-fostering zebra finch eggs with Bengalese finch pairs. We repeatedly sampled the gut microbiota of juveniles at different developmental stages, from hatchling to adulthood, and characterised the gut microbial profiles using 16S ribosomal RNA (rRNA) gene amplicon sequencing. To understand the relative strengths of dispersal from the social environment and host selection, we evaluated microbial similarity between the fostered juveniles and their genetic relatives as well as their foster relatives at different ontogenetic stages.

## Materials and methods

### Study organisms and experimental design

We conducted these experiments between January and August 2017 on two captive estrildid finch species, zebra finches and Bengalese finches, from the laboratory stock at Bielefeld University.

**Fig. 1:**
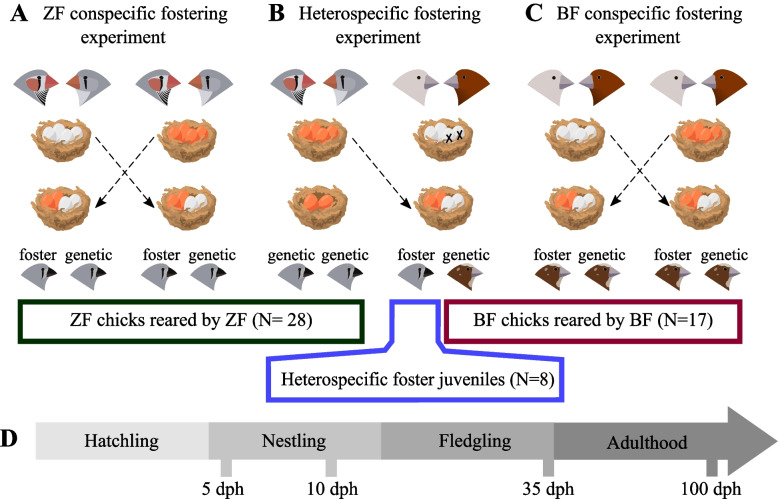
Experimental design. In the conspecific cross-fostering experiments with **A** zebra finches (ZF) and **C** Bengalese finches (BF), the second and third eggs were swapped between conspecific nests. **C** In the heterospecific cross-fostering experiment, the second and third eggs from ZF nests were transferred into BF nests after removing the second and third BF eggs. We assessed the microbial similarity between (i) age groups, (ii) different sample types and (iii) genetic vs. foster relatives. **D** Sampling scheme over bird ontogeny: 5, 10, 35 and 100 days post-hatch (dph). This figure was created by Omar Castillo-Gutiérrez using bird illustrations designed by Sonja Engel, the copyright holder

Initially, males and females that were not genetically related were transferred from indoor aviaries (2.30 × 2.90 × 3.30 m) to indoor cages (0.80 × 0.30 × 0.40 m) in male-female pairs to generate 42 breeding pairs of zebra finches and 22 pairs of Bengalese finches. After a habituation period of 1 week, all cages were provided with coconut fibre nesting material and a wooden nest box (15 × 15 × 15 cm). During daily nest checks, nest construction was monitored, and freshly laid eggs were marked with an odourless permanent marker to distinguish the laying order. Older eggs were candled with a flashlight to detect signs of fertilisation. We excluded 27 zebra finch (ZF) pairs and 15 Bengalese finch (BF) pairs that failed to breed successfully (no nest or fertilised eggs) or due to the lack of compatible foster counterparts (i.e. a nest containing fertilised eggs at a comparable developmental stage). In total, we used 15 ZF pairs and 7 BF pairs. These pairs were assigned to one of the three cross-fostering experiments: the ZF conspecific experiment (where we cross-fostered eggs between two unrelated ZF nests, Fig. 1A), the heterospecific experiment (where we cross-fostered half of the eggs of a ZF clutch to BF nests, Fig. 1B) and the BF conspecific experiment (where we cross-fostered eggs between two unrelated BF nests, Fig. 1C). However, two BF pairs that had two successive clutches were used twice, with each clutch assigned to a different experiment (one to the heterospecific experiment, the other to the BF conspecific experiment).

We investigated the relative impact of within-species genetic differences and the social environment in the two conspecific cross-fostering experiments, where we cross-fostered eggs between conspecific nests (ZF conspecific cross-fostering experiment; *N*_NEST_ = 10, Fig. 1A; BF conspecific cross-fostering experiment; *N*_NEST_ = 4, Fig. 1C). We swapped the second and third eggs between two compatible conspecific clutches, while the rest of the eggs remained in their genetic nests (Fig. 1A, C). In these experiments, the parents reared the juveniles of genetically unrelated conspecifics (*N*_ZF JUVENILE_ = 11; *N*_BF JUVENILE_ = 6) along with their genetic juveniles (*N*_ZF JUVENILE_ = 9; *N*_BF JUVENILE_ = 4).

In the heterospecific cross-fostering experiment, we investigated the influence of the social environment and species-specific factors on the gut microbiota by cross-fostering eggs from ZF nests (*N*_NEST_ = 5) into compatible BF nests (*N*_NEST_ = 5). We transferred the second and third eggs of a ZF clutch into a BF nest and removed the second and third eggs of the BF clutch to maintain the initial clutch size (Fig. 1B); the rest of the BF eggs stayed in the natal nest. As a result, ZF adults reared their genetic juveniles (*N* = 8), while BF adults reared ZF juveniles (i.e. ZF juveniles fostered by BF adults, hereafter heterospecific foster juveniles, *N* = 8) along with their genetic juveniles (*N* = 7; Fig. 1B).

During the daily nest checks, we labelled the freshly hatched chicks by cutting their down feathers on different areas of the body [64]. The social families (i.e. the adults and their genetic and foster juveniles) were kept together in the breeding cages until the youngest juvenile in the clutch reached nutritional independence (approximately 35 dph). After this point, the adult birds were transferred into mixed-sexed indoor aviaries and kept with other conspecifics from this study. All the juveniles were moved to single-species aviaries containing juveniles and two adult tutor birds. We are aware that this relocation allowed transmission of microbes between heterospecific foster juveniles and conspecifics in the same aviary, hindering assessment of the relative importance of host selection and dispersion (see the “Sampling” section). However, this relocation was necessary, as juveniles can only learn their species-specific song from conspecifics and only during the sensitive period for acoustic learning, which is between 25 and 90 dph in zebra finches [65].

All birds were monitored daily until the last sampling, which occurred when the youngest juvenile in the clutch was approximately 100 dph. During the whole experimental period, birds were kept under a 14 h:10 h light/dark cycle (in addition to natural light conditions) in a temperature range of 24.5–25.5°C. Each day, they received a standard diet containing seeds, germinated seeds ad libitum, a vitamin-mineral supplement and additional egg food (Tropical Finches, CéDé, Evergreen, Belgium). All birds were kept in our aviary stock at Bielefeld University.

### Sampling

We investigated the microbial community profiles from faeces, a reliable proxy for the gut microbiota [66, 67]. We collected faeces from adults and juveniles at four different sampling times. The first and second samples were collected during the nestling period when the youngest juvenile in the nest was 5 and 10 dph, respectively (Fig. 1D). The third sample was collected when the youngest juvenile was 35 dph, i.e. when the juveniles had reached nutritional independence. We collected the fourth sample after all the offspring reached sexual maturity, when the youngest offspring reached 100 dph. However, as the heterospecific foster juveniles were transferred into aviaries with conspecifics after 35 dph, we did not include samples collected from these juveniles at 100 dph (*N*=8) in any statistical analyses (see “Study organisms and experimental design” section). These samples were only used to assess general patterns in the microbial communities. We sampled all the parents and juveniles that survived until 100 dph. In total, we collected 396 samples from 95 individuals (see Additional file 1 for the detailed sample size).

To obtain faecal samples from the 5- and 10-day-old juveniles, we placed them on a sterile aluminium plate under a heat lamp for 10 min. To sample the 35- and 100-day-old juveniles and adults, we placed the individual birds in a sampling cage (30 × 40 × 30 cm) with the ground covered by a sterile aluminium plate for approximately 30 min. We transferred the faecal materials into 1.5-ml Eppendorf tubes, placed them immediately on ice and stored them at −80°C until further processing. All sampling procedures were performed under sterile conditions.

### DNA extraction and library preparation

We extracted microbial DNA from 0.02 g of the faecal sample using the QIAamp PowerFecal DNA Kit (Qiagen, Germany) following the manufacturer’s instructions. The 16S rRNA gene libraries were prepared following the Illumina 16S Metagenomic Library Preparation Guide 15044223-B. The protocol details have been previously described in Maraci et al. [38]. In short, we targeted the hypervariable V3–V4 regions of the 16S rRNA gene by performing two-step polymerase chain reactions (PCRs). The amplification success was evaluated on a Bioanalyzer DNA 1000 chip (Agilent Technologies, Palo Alto, CA, USA), and libraries with low concentrations were excluded (*N*=13). In addition to the biological samples, the final library pool contained five negative controls for the sampling, extraction, PCR and clean-up steps. The final library was sequenced using paired-end mode (2 × 300 sequencing cycles) on the Illumina MiSeq system (Illumina, Inc., San Diego, CA, USA) at CeBiTec, Bielefeld University.

### Data analyses

Bioinformatics processing was performed as described in Engel et al. [39]. In short, the MiSeq PE reads were assembled in an iterative manner using Flash v1.2.11 [68]. We performed adapter clipping using cutadapt v1.18 [69]; dereplication, alignment, filtering and denoising using mothur v1.41.3 [70]; chimaera checking and OTU clustering with an identity threshold of 97%, using USEARCH v8.0.1477 [71]; and taxonomic classification based on the full SILVA database v138 [72].

All statistical analyses were carried out in R v4.0.0 [73] and Primer-e software v7 [74]. As an initial filtering step, samples with less than 10,000 total read counts (*N*=8) were discarded from the dataset. Additionally, we excluded all OTUs that could not be classified at the phylum level (*N*=1) and that were classified as mitochondria or chloroplasts (*N* = 59) as well as all singletons (*N*=79) are likely to be issued by sequencing errors.

After the filtering steps, we rarefied OTU read-count data to the lowest read count observed in the dataset and calculated alpha diversity metrics, i.e. Shannon’s diversity index, which considers both the abundance and evenness of the taxa present [75], and Faith’s phylogenetic diversity, which incorporates phylogenetic relationships between microbial taxa [76]. We fitted LMMs using these two indices as the response variables, sample type (i.e. ZF adults, ZF juveniles, BF adults, BF juveniles or heterospecific foster juveniles) and sampling time (i.e. 5, 10, 35 or 100 dph) as fixed effects using the *lme4* package v1.1−15 [77]. We also included the rearing nest as a random factor to account for the nonindependence of individuals that shared a nest. In addition, we performed pairwise testing (between sample types for each sampling time and between sampling times for each sample type) using the *Multicomp* package [78]. The composition of the microbial communities across different sample types was visualised using stacked-bar plots based on the family-level taxonomy using *ggplot2* v3.3.2 [79].

To estimate between-group differences, we applied Cumulative Sum Scaling (CSS) normalisation [80] using the r package *metagenomeseq* v1.30.0 [81] to the filtered dataset. Subsequently, we calculated the dissimilarity matrix based on BC dissimilarities [82] and WU distances [83]. We assessed compositional differences among sample types with a PERMANOVA [84] with 9999 permutations using Primer-e. We also visualised the dissimilarities among sample types at each sampling time using nMDS based on BC dissimilarities with Primer-e. We identified differentially abundant OTUs among sampling times using the *Corncob* package [85], a method developed explicitly for microbial differential analysis. It estimates taxa-specific differential abundances by building beta-binomial regression models, controlling for differential variability across the covariate of interest. We conducted this method using nonrarefied data and set the significance threshold for *p* values to 0.05 after Benjamini and Hochberg FDR correction [86].

We also explored whether the gut microbiota of the foster juveniles was more similar to their genetic relatives or foster relatives at each sampling time. We first extracted pairwise dissimilarity indices from the BC dissimilarity matrix for the following six groups: foster juvenile and their genetic relatives (genetic mothers, fathers and siblings; all remained in the natal nest and had no physical contact with the foster juvenile) as well as foster juvenile and their foster relatives (foster mothers, fathers and siblings). The dissimilarity index ranges from 0 to 1, where samples with identical communities scored 0 and samples with the greatest differences scored 1. Then, we assessed whether there was a difference in the microbial dissimilarity between foster juveniles and all genetic relatives versus foster relatives at each sampling time using Wilcoxon rank-sum tests. Subsequently, we compared the microbial dissimilarity at each sampling time among the following groups: (i) foster juvenile and their genetic mothers versus foster juvenile and their foster mothers, (ii) foster juvenile and their genetic fathers versus foster juvenile and their foster father and (iii) foster juvenile and their genetic siblings versus foster juvenile and their foster siblings. Then, we assessed whether these distances changed over time using the Kruskal–Wallis rank-sum test followed by post hoc Dunn’s test with a Bonferroni correction.

We estimated the relative contributions of genetic relatives (genetic mothers, fathers and siblings that had no social contact with the foster juveniles) and foster relatives (foster mothers, fathers, and siblings that shared the same social environment) to the gut microbiota of heterospecific foster juveniles based on the SourceTracker analysis [87]. This method uses a Bayesian approach to estimate the proportion of OTUs in a given community that originate from a potential source environment.

Furthermore, we identified microbial OTUs that preferentially occurred in a given sample type or age group by indicator species analysis using the *multipatt* function in the *indicspecies* package [88]. In this method, an IndVal for each microbial taxon is inferred by placing the relative abundance of a taxon in a given group in context with its occurrence in an entire community. The IndVal is calculated as the product of specificity and fidelity values ranging between 0 and 1, where 1 indicates taxon exclusivity in a given group.

## Results

We investigated how gut microbial communities change over host ontogeny and how social transmission and host selection influence the establishment of the gut microbiota in zebra finches and Bengalese finches. We conducted three cross-fostering experiments under controlled conditions (Fig. 1): (i) in the zebra finch (ZF) conspecific experiment, we cross-fostered eggs between two unrelated ZF pairs (Fig. 1A); (ii) in the Bengalese finch (BF) conspecific experiment, we cross-fostered the eggs between two unrelated BF pairs (Fig. 1C); and in the heterospecific experiment, we fostered half of the eggs from a ZF clutch to BF pairs (Fig. 1B). In all experiments, we sampled the juveniles and their parents when the youngest juvenile in the nest was 5, 10, 35 and 100 days post-hatch (dph) (Fig. 1D). Employing 16S ribosomal RNA gene sequencing, we first documented ontogenetic changes in the gut microbiota of ZF and BF juveniles reared by conspecifics. Second, we investigated whether microbial communities of juveniles fostered to conspecifics (i.e. ZF and BF juveniles reared by unrelated conspecifics) and juveniles fostered to heterospecifics (i.e. ZF juveniles reared by BF adults) were more similar to their genetic or foster relatives.

After filtering, our dataset contained 808 operational taxonomic units (OTUs) across 375 samples with an average read count of approximately 71,330 (minimum = 4033; maximum = 461,885; SD = 47,046.12). In total, we identified 20 microbial phyla, with *Firmicutes* (67.4%, SD = 27.4%), *Campilobacterota* (22.4%, SD = 25.3%), *Proteobacteria* (6.2%, SD = 13.9%) and *Actinobacteria* (4.0%, SD = 8.7%) being the most prevalent. The identified microbial taxa corresponded to 231 microbial families, but only the following five had a mean abundance higher than 1%: *Lactobacillaceae* (62.%, SD = 29.2%), *Campylobacteraceae* (21.9%, SD = 25.1%), *Enterobacteriaceae* (4.7%, SD = 12.3%), *Leuconostocaceae* (2.8%, SD = 5.9%), *Bifidobacteriaceae* (2.6%, SD = 6.7%) and *Enterococcaceae* (1.2%, SD = 4.0%).

### Ontogenetic changes in the gut microbiota

To investigate how the gut microbiota of juveniles changes over time, we generated two datasets, each containing samples from ZF or BF juveniles reared by conspecifics at 5, 10, 35 and 100 dph. Based on our former study in the same species, the microbial diversity fluctuates during the different phases of the breeding period, particularly in males, probably due to hormonal fluctuations [38]. Therefore, we included adult samples collected only during the post-breeding period (i.e. 100 days after the youngest juvenile in a clutch hatched, where the juveniles are considered to reach sexual maturity and parents are not in the breeding period), considering these samples provide a more accurate representation of adult-state gut microbiota.

#### Alpha diversity

**Fig. 2:**
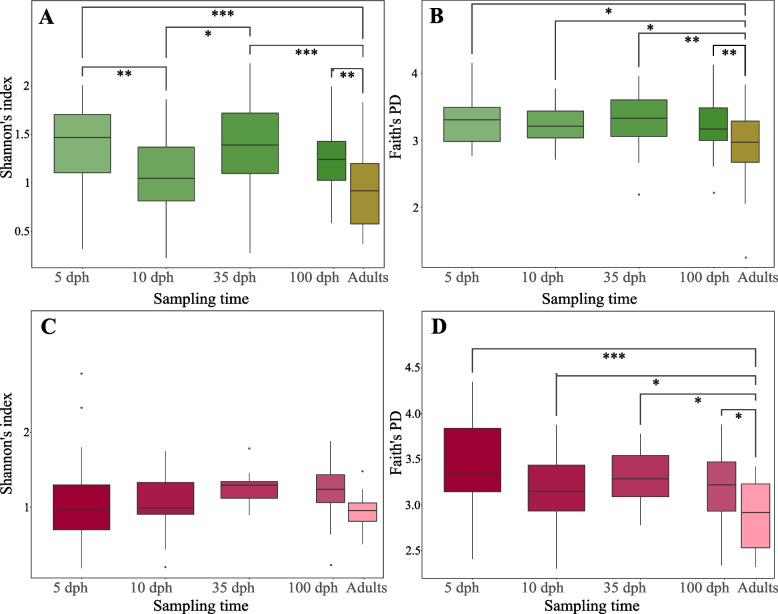
Ontogenetic changes in alpha diversity. The changes in **A** Shannon’s diversity index across zebra finch ontogeny, **B** Faith’s phylogenetic diversity index across zebra finch ontogeny, **C** Shannon’s diversity index across Bengalese finch ontogeny and **D** Faith’s phylogenetic diversity index across Bengalese finch ontogeny. The only adult samples included in this analyses are the ones collected at the post-breeding stage (i.e. when their juveniles reach sexual maturity). The significant differences were determined based on the linear mixed model at *p* values ≤ 0.05 (*), *p* ≤ 0.01 (**) and *p* ≤ 0.001 (***). The lines within the box plots indicate the medians, and the lower and upper boundaries of the boxes indicate the 25th and 75th percentiles, respectively. Whiskers above and below the boxes correspond to 1.5 times the interquartile range (IQR) above and below the 25th and 75th percentiles, respectively

Shannon’s diversity index differed significantly among the juvenile ZF age groups (linear mixed model (LMM); *R*^2^-marginal=0.135, *R*^2^-conditional=0.263; Fig. 2A, see also Additional file 2 for pairwise comparisons), while there was no significant alteration in Faith’s phylogenetic diversity index (Fig. 2B, see also Additional file 2 for pairwise comparisons). Based on both metrics, juvenile ZFs consistently exhibited higher alpha diversity than adults at the post-breeding stage, as well as at 100 dph (Fig. 2A, B). In contrast, Shannon’s diversity index did not differ among the juvenile age groups or between BF juveniles and adults at the post-breeding stage (Fig. 2C), while Faith’s phylogenetic diversity index was consistently higher in juveniles than in adults at the post-breeding stage (LMM; *R*^2^-marginal=0.129, *R*^2^-conditional=0.217; Fig. 2D, see Additional file 3).

#### Beta diversity

We found significant group differences in community composition across ontogenetic stages in ZF and BF juveniles based on both Bray–Curtis (BC) and weighted UniFrac (WU) distance (see Table 1 for main permutational multivariate analysis of variance (PERMANOVA) models and the pairwise comparisons). ZF juveniles exhibited different gut microbiota from adults at all sampling times, including 100 dph, based on both metrics (see Additional file 4 for nMDS plots). BF juveniles and adults also exhibited differential microbial profiles at all sampling times based on both metrics, with the exception of 100 dph samples based on the WU distance (see Additional file 5 for nMDS plots).

**Table 1:** PERMANOVA results based on BC dissimilarity and WU distances between different age groups. *P* values less than 0.05 are shown in bold

**Zebra finches**				
**Main model**	**BC**		**WU**	
	***F***	***p***	***F***	***p***
	**2.8812**	**0.0001**	**2.4215**	**0.0001**
**Pairwise comparisons**	**BC**		**WU**	
**Groups**	***t***	***p***	***t***	***p***
ZF juvenile at day 5 vs ZF adults	**2.001**	**0.0001**	**2.0419**	**0.0003**
ZF juvenile at day 10 vs ZF adults	**2.032**	**0.0001**	**1.7338**	**0.0051**
ZF juvenile at day 35 vs ZF adults	**2.007**	**0.0002**	**1.5525**	**0.0173**
ZF juvenile at day 100 vs ZF adults	**1.371**	**0.0141**	**1.4408**	**0.0311**
ZF juvenile at day 5 vs ZF juvenile at day 10	1.090	0.2158	0.9074	0.5666
ZF juvenile at day 5 vs ZF juvenile at day 35	**1.521**	**0.0014**	**1.4793**	**0.0255**
ZF juvenile at day 5 vs ZF juvenile at day 100	**1.705**	**0.0002**	**1.868**	**0.0014**
ZF juvenile at day 10 vs ZF juvenile at day 35	**1.620**	**0.0004**	1.2525	0.109
ZF juvenile at day 10 vs ZF juvenile at day 100	**1.797**	**0.0001**	**1.6676**	**0.0037**
ZF juvenile at day 35 vs ZF juvenile at day 100	**1.509**	**0.0029**	1.2875	0.0971
**Bengalese finch**				
**Main model**	**BC**		**WU**	
	***F***	***p***	***F***	***p***
	**3.1828**	**0.0001**	**3.4155**	**0.0001**
**Pairwise comparisons**	**BC**		**WU**	
**Groups**	***t***	***p***	***t***	***p***
BF juvenile at day 5 vs adults	**2.1535**	**0.0001**	**2.3876**	**0.0004**
BF juvenile at day 10 vs adults	**2.134**	**0.0001**	**1.8493**	**0.0043**
BF juvenile at day 35 vs adults	**2.3217**	**0.0001**	**2.3091**	**0.0011**
BF juvenile at day 100 vs adults	**1.437**	**0.0113**	1.3664	0.0698
BF juvenile at day 5 vs BF juvenile at day 10	1.0995	0.2054	1.1981	0.1552
BF juvenile at day 5 vs BF juvenile at day 35	**1.5001**	**0.0013**	**1.6218**	**0.0152**
BF juvenile at day 5 vs BF juvenile at day 100	**1.9234**	**0.0001**	**2.2347**	**0.0005**
BF juvenile at day 10 vs BF juvenile at day 35	**1.3442**	**0.0163**	1.2775	0.1037
BF juvenile at day 10 vs BF juvenile at day 100	**1.7904**	**0.0002**	**1.7385**	**0.0097**
BF juvenile at day 35 vs BF juvenile at day 100	**1.9065**	**0.0001**	**2.1895**	**0.0007**

**Fig. 3:**
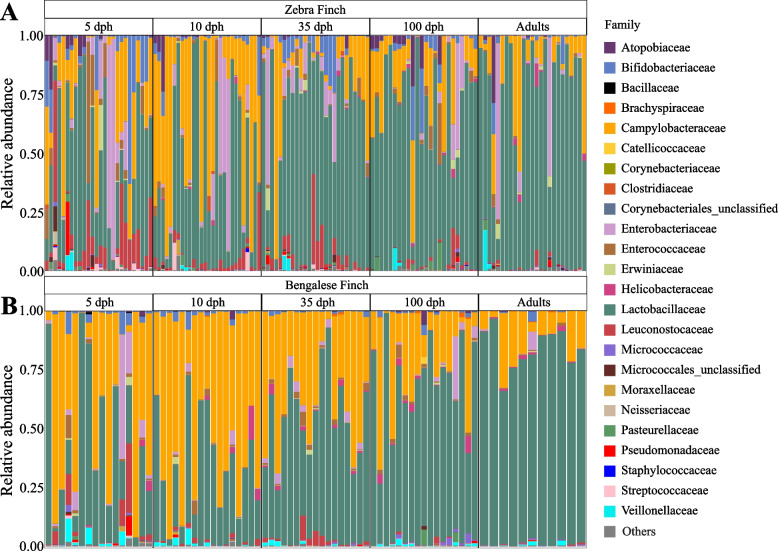
Relative abundance of microbial families in gut samples. The 20 most abundant microbial families in juvenile **A** zebra finches and **B** Bengalese finches throughout different developmental stages are shown. The remaining microbial families are submerged as Other

The gut microbiota underwent substantial compositional changes during host ontogeny in both species. In zebra finches, the family *Lactobacillaceae* dominated the community at all sampling times, yet its mean abundance increased from 38.09% (SD=26.46%) to 65.74% (SD=24.91%) between 5 and 100 dph, exhibiting the highest mean abundance in adults (74.45%, SD=27%) (Fig. 3A, Additional file 6). In contrast, the families *Campylobacteraceae, Leuconostocaceae, Enterobacteriaceae, Bifidobacteriaceae* and *Enterococcaceaea* had a lower relative abundance in adults (Fig. 3A, Additional file 6). In Bengalese finches, *Campylobacteraceae* was the predominant microbial family at 5 and 10 dph but exhibited a marked decrease in adults (Fig. 3B, Additional file 7). *Lactobacillaceae* became the dominant microbial family at 35 dph. Similar to zebra finches, the mean abundances of the families *Enterobacteriaceae*, *Leuconostocaceae* and *Bifidobacteriaceae* decreased with juvenile development (Fig. 3B, Additional file 7).

**Fig. 4:**
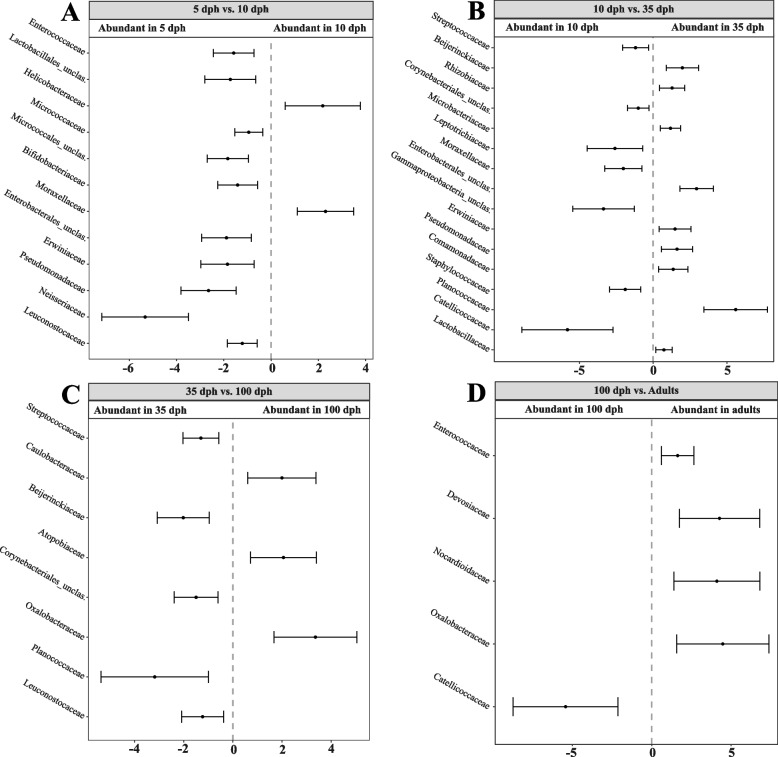
Differentially abundant OTUs at different ontogenetic stages in zebra finches. Differentially abundant OTUs between **A** samples at 5 and 10 dph, **B** samples at 10 and 35 dph and **C** samples at 35 and 100 dph from juvenile zebra finches as well as **D** samples at 100 dph and adult zebra finches were determined using beta-binomial regression models in the *Corncob* package. The family-level taxonomy of each corresponding OTU is shown

**Fig. 5:**
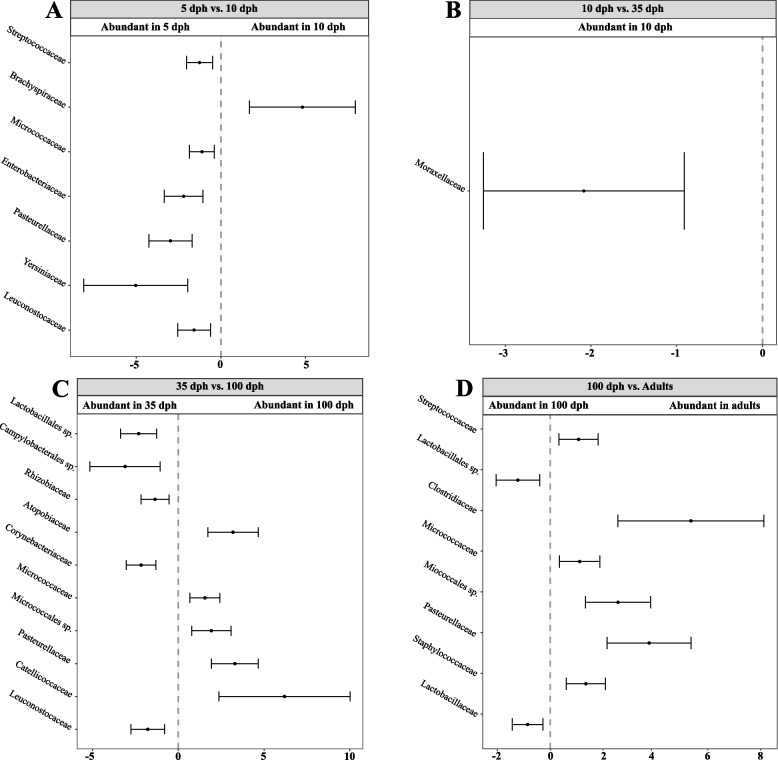
Differentially abundant OTUs among different ontogenetic stages in Bengalese finches. Differentially abundant OTUs between **A** samples at 5 and 10 dph, **B** samples at 10 and 35 dph and **C** samples at 35 and 100 dph in juvenile Bengalese finches as well as **D** samples from 100 dph juveniles and adult Bengalese finches were determined using beta-binomial regression models in the *Corncob* package. The family-level taxonomy of each corresponding OTU is shown

To better understand how the gut microbiota changes over ontogeny, we identified differentially abundant OTUs between samples collected at different times using beta-binomial regression models and controlling for differential variability across the covariates of interest using the *Corncob* package [85]. We identified 12 differentially abundant OTUs between the 5 and 10 dph samples of ZF juveniles, 10 of which were more abundant in the 5 dph samples (Fig. 4A). Samples at 10 dph and 35 dph showed the largest number of differentially abundant OTUs, where seven and nine OTUs were more abundant in the 10 and 35 dph samples, respectively (Fig. 4B). Finally, a comparison of juvenile samples at 100 dph and adult samples showed only five differentially abundant OTUs (Fig. 4D). One OTU classified as the family *Oxalobacteraceae* was more abundant in juveniles at 100 dph, while four OTUs belonging to the families *Enterococcaceae*, *Devosiaceae*, *Nocardioidaceae* and *Catellicoccaceae* were more abundant in adults. In Bengalese finches, we identified seven differentially abundant OTUs between the 5 dph and 10 dph samples (Fig. 5A). Among these, one OTU of the *Brachyspiraceae* family was significantly more abundant at 10 dph. Notably, when comparing OTU abundances between juveniles at 10 and 35 dph, we found only one differentially abundant OTU at 10 dph: the *Moraxellaceae* family (Fig. 5B). Most differentially abundant OTUs were identified when comparing 35 and 100 dph samples (Fig. 5C), indicating that several microbial taxa became less abundant during this period while others were obtained. A comparison between juvenile samples at 100 dph and adult samples yielded eight differentially abundant OTUs, of which only two were more abundant in juveniles.

### Microbial similarity between conspecifics increases as development progresses

The heterospecific cross-fostering experiment aimed to disentangle the influence of social factors and host-specific factors on the development of the gut microbiota. Therefore, we compared the gut microbiota of heterospecific foster juveniles, that of ZF juveniles and BF juveniles raised by conspecifics and that of the adults of both species. For this analysis, we excluded the 100 dph samples from heterospecific foster juveniles (see the Sampling section). The 100 dph comparisons were only conducted for the juvenile groups raised by conspecifics.

#### Alpha diversity

The alpha diversity of heterospecific foster juveniles did not differ significantly from those of zebra finch juveniles and Bengalese finch juveniles raised by their conspecifics, or that of the adults of both species or that of ZF juveniles and BF juveniles raised by conspecifics, except for at 35 dph: at this sampling time, heterospecific foster juveniles had the highest Shannon diversity, which significantly differed from that of the adults of both species (see Additional file 8 for pairwise comparisons and 9 for alpha diversity plots). Here, it should be noted that the lack of a statistical difference in alpha diversity might originate from our relatively small sample size for this group (*N*=8).

#### Beta diversity

**Fig. 6:**
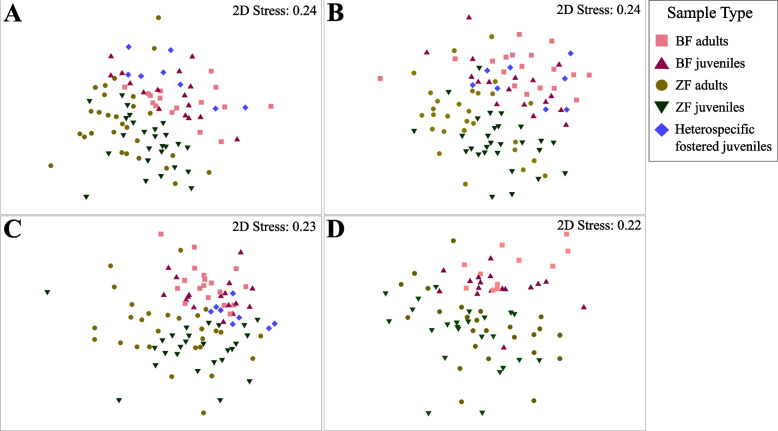
nMDS plots of the dissimilarities of the gut microbiota among sample types. Samples collected at **A** 5 dph, **B** 10 dph, **C** 35 dph and **D** 100 dph. Distances were computed using the BC dissimilarity index

When visualising the microbial resemblance among sample types (i.e. ZF juveniles and adults, BF juveniles and adults, and heterospecific foster juveniles) at different sampling times using nonmetric multidimensional scaling (nMDS) based on BC dissimilarity, we observed that conspecifics had more similar microbial profiles at all sampling times, except for heterospecific foster juveniles. These samples were more similar to the samples collected from BF juveniles at 5 and 10 dph (Fig. 6A, B). At 35 dph, the samples originating from heterospecific foster juveniles clustered together at the intersection between ZF and BF samples (Fig. 6C). By 100 dph, the distance between BF and ZF juveniles had increased, indicating that the microbial composition becomes more species-specific as development progresses (Fig. 6D).

The PERMANOVA detected differences among the sample types (BC dissimilarity: F=10.86, *p*<0.001; WU distance: F= 8.23, *p*<0.001) over time (BC dissimilarity: F=4.83, *p*<0.001; WU distance, F= 4.36, *p*<0.001) as well as an interaction between these two factors (BC dissimilarity: F=1.73, *p*<0.001; WU distance: F= 1.72, *p*<0.001). By conducting pairwise comparisons of dissimilarities among the different sample types at each sampling time, we observed significant differences between zebra finches and Bengalese finches at all sampling times (Table 2). The samples originating from heterospecific foster juveniles differed from ZF adults and juveniles at all sampling times (Table 2). A comparison of the heterospecific foster juveniles with and BF juveniles revealed that these two groups differed according to their BC dissimilarity, but not WU distance at 5 dph (Table 2). At 10 dph, neither of these metrics exhibited significant group differences; however, at 35 dph, heterospecific foster juveniles significantly differed from BF juveniles (Table 2).

**Table 2:** Pairwise PERMANOVA results based on BC dissimilarity and WU distances between different sample types. *P* values less than 0.05 are shown in bold

Sample type	5 dph		10 dph		35 dph		100 dph	
	***t***	***p***	***t***	***p***	***t***	***p***	***t***	***p***
**Pairwise comparisons based on BC dissimilarity**								
BF adults *vs* ZF adults	**2.25**	**0.0001**	**2.56**	**0.0001**	**2.35**	**0.0001**	**1.37**	**0.0137**
BF juveniles *vs* ZF juveniles	**2.25**	**0.0001**	**2.23**	**0.0001**	**2.22**	**0.0001**	**2.16**	**0.0001**
BF juveniles *vs* BF adults	**1.52**	**0.0065**	**1.81**	**0.0001**	**1.69**	**0.0003**	**1.44**	**0.0116**
BF juveniles *vs* ZF adults	**2.18**	**0.0001**	**2.24**	**0.0001**	**2.13**	**0.0001**	**2.17**	**0.0001**
ZF juveniles *vs* ZF adults	**1.74**	**0.0001**	**1.79**	**0.0002**	**1.57**	**0.0009**	**2.23**	**0.0001**
ZF juveniles *vs* BF adults	**2.40**	**0.0001**	**2.90**	**0.0001**	**2.73**	**0.0001**	**2.37**	**0.0001**
Heterospecific foster juveniles *vs* BF adults	1.29	0.0581	**1.44**	**0.0053**	**2.02**	**0.0001**	NA	NA
Heterospecific foster juveniles *vs* BF juveniles	**1.36**	**0.0313**	1.19	0.0874	**1.51**	**0.0038**	NA	NA
Heterospecific foster juveniles *vs* ZF adults	**1.90**	**0.0001**	**1.98**	**0.0001**	**2.02**	**0.0001**	NA	NA
Heterospecific foster juveniles *vs* ZF juveniles	**2.11**	**0.0001**	**2.01**	**0.0001**	**1.75**	**0.0004**	NA	NA
**Pairwise comparisons based on WU distance**								
BF adults *vs* ZF adults	**2.36**	**0.0001**	**2.41**	**0.0001**	**2.23**	**0.0001**	**2.24**	**0.0001**
BF juveniles *vs* ZF juveniles	**1.64**	**0.0118**	**1.65**	**0.0082**	**2.18**	**0.0001**	**2.30**	**0.0001**
BF juveniles *vs* BF adults	**1.63**	**0.013**	1.40	0.0610	1.33	0.0804	1.37	0.0694
BF juveniles *vs* ZF adults	**1.63**	**0.0069**	**1.65**	**0.0090**	1.31	0.0755	**2.14**	**0.0004**
ZF juveniles *vs* ZF adults	**1.68**	**0.0052**	**1.57**	**0.0196**	**2.03**	**0.0004**	**1.44**	**0.0291**
ZF juveniles *vs* BF adults	**2.03**	**0.0001**	**2.40**	**0.0002**	**2.54**	**0.0001**	**2.47**	**0.0001**
Heterospecific foster juveniles *vs* BF adults	**1.52**	**0.0312**	1.12	0.2374	**2.05**	**0.0021**	NA	NA
Heterospecific foster juveniles *vs* BF juveniles	1.23	0.1398	0.85	0.7058	**1.65**	**0.0158**	NA	NA
Heterospecific foster juveniles *vs* ZF adults	**1.63**	**0.0048**	**1.57**	**0.0102**	**1.83**	**0.0012**	NA	NA
Heterospecific foster juveniles *vs* ZF juveniles	**1.69**	**0.0063**	**1.42**	**0.0388**	**1.61**	**0.0178**	NA	NA

**Fig. 7:**
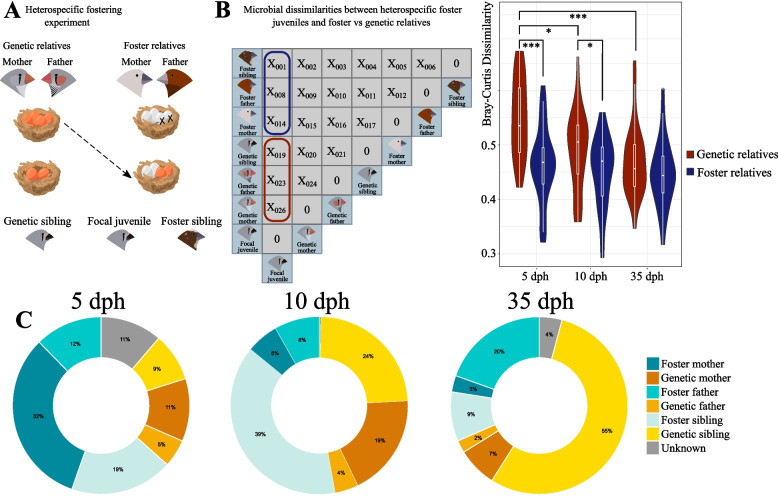
Microbial similarities/dissimilarities of heterospecific foster juveniles with their genetic and foster families. **A** Heterospecific cross-fostering experiment. **B** *Pairwise dissimilarity matrix* (left) based on Bray–Curtis dissimilarities, illustrated for one bird. The numbers illustrated as X are the dissimilarity index, and the actual values range from 0 to 1, where samples with identical communities scored 0 and samples with the greatest differences scored 1. *Violin plots* (right) with embedded box plots comparing the distribution of pairwise dissimilarities between heterospecific foster juveniles and their genetic relatives (genetic mothers, fathers and siblings) and foster relatives (foster mothers, fathers and siblings) at 5, 10 and 35 dph. Significance differences between foster and genetic relatives were determined based on Wilcoxon rank-sum tests. The significance of the change in distance over time between the foster and genetic groups was determined by the Kruskal–Wallis rank-sum test followed by the post hoc Dunn’s test. Significance is highlighted for *p* values ≤ 0.05 (*), *p* ≤ 0.01 (**) and *p* ≤ 0.001 (***) after Bonferroni correction. **C** Estimated proportions of OTUs in the gut microbiota of heterospecific foster juveniles originating from genetic and foster relatives at 5, 10 and 35 dph based on SourceTracker2 analyses. This figure was created by Omar Castillo-Gutiérrez using bird illustrations designed by Sonja Engel, the copyright holder

Next, we investigated whether the microbial communities of heterospecific foster juveniles were more similar to those of their genetic relatives or foster relatives using a distance-based approach (Fig. 7A). We compared the pairwise BC dissimilarity between heterospecific foster juveniles and their genetic relatives (genetic mother, father and siblings) with the pairwise distance between heterospecific foster juveniles and their foster relatives (foster mother, father and siblings) with a Wilcoxon rank-sum exact test. We found that at 5 dph (*p*<0.001) and at 10 dph (*p*=0.027), the microbial distance between heterospecific foster juveniles and their genetic relatives was higher than that between heterospecific foster juveniles and their foster relatives (Fig. 7B). However, at 35 dph, there was no difference between these groups. Similarities between juveniles and their foster relatives did not change over time. However, heterospecific foster juveniles became more similar to their genetic relatives over time (Kruskal–Wallis rank-sum test, *p*<0.001), with significant differences in the similarity between 5 and 10 dph (post hoc Dunn’s test, *p*=0.027), as well as 5 dph and 35 dph (post hoc Dunn’s test, *p*<0.001) (Fig. 7B). To determine whether similarities between heterospecific foster juveniles and specific foster or genetic relatives (mother, father or sibling) change over time, we compared the dissimilarities for the following paired groups: (i) juvenile and their genetic mothers versus juvenile and their foster mothers, (ii) juvenile and their genetic fathers versus and their foster fathers and (iii) juvenile and their genetic siblings versus juvenile and their foster siblings. We did not observe any differences in the microbial similarity between heterospecific foster juveniles and their foster and genetic parents (both mothers and fathers) within each sampling point, as the distance between these paired groups did not change over time. Nevertheless, at 5 dph, the microbial dissimilarity between heterospecific foster juveniles and their genetic siblings was significantly higher than that between heterospecific foster juveniles and their foster siblings (Wilcoxon rank-sum exact test, *p*<0.001); this difference was not evident at other sampling times. Furthermore, there was no change in the microbial dissimilarity between these juveniles and their foster siblings over time. In contrast, the distance between heterospecific foster juveniles and their genetic siblings decreased over time (Kruskal–Wallis rank-sum test, *p*<0.001), with a significant difference between 5 and 10 dph (post hoc Dunn’s test, *p*=0.016) as well as 5 and 35 dph (post hoc Dunn’s test, *p*<0.001).

We also estimated the relative contributions of genetic and foster relatives to the proportions of OTUs in the gut microbiota of heterospecific foster juveniles at 5, 10, and 35 dph using SourceTracker2. At 5 dph, an average of 63% of OTUs originated from foster relatives (Fig. 7C). The highest proportion of OTUs came from foster mothers (32%), and the contributions of foster fathers and foster siblings were 12 and 19%, respectively. On average, only 25% of OTUs were concordant to genetic relatives (5% from genetic fathers, 11% from genetic mothers and 9% from genetic siblings). At 10 dph, the relative contributions of foster and genetic relatives were 53 and 47%, respectively, and the dominant source of the nestling gut microbiota was foster siblings, which contributed approximately 39%. At 35 dph, the proportion of OTUs originating from foster relatives decreased to 32%, while the proportion of OTUs sourced from genetic relatives increased to 64%, with the predominant source being the gut microbiota of genetic siblings (at 54%) (Fig. 7C).

We also identified ten indicator OTUs for each sample type using indicator value (IndVal) analyses (Additional file 10). Strikingly, seven of these indicator OTUs were specific to heterospecific foster juveniles; five were indicators for samples at 5 dph, and some of these belonged to potentially pathogenic microbial genera such as *Streptococcus* and *Corynebacterium* (Additional file 10). Interestingly, the indicator OTUs for samples collected from heterospecific foster juveniles at 35 dph belong to the genus *Paenibacillus*, a widely used probiotic in poultry to improve the immune condition [89].

### Decreases in the microbial similarity between juveniles and their conspecific foster relatives over development

We investigated the influence of intraspecific selection mechanisms and social transmission on the establishment of the gut microbiota using two sets of conspecific cross-fostering experiments. In these experiments, we cross-fostered eggs between the nests of unrelated conspecifics in both zebra finches and Bengalese finches. We compared BC dissimilarity between the paired groups of foster and genetic relatives for each experimental group at 5, 10, 35 and 100 dph.

**Fig. 8:**
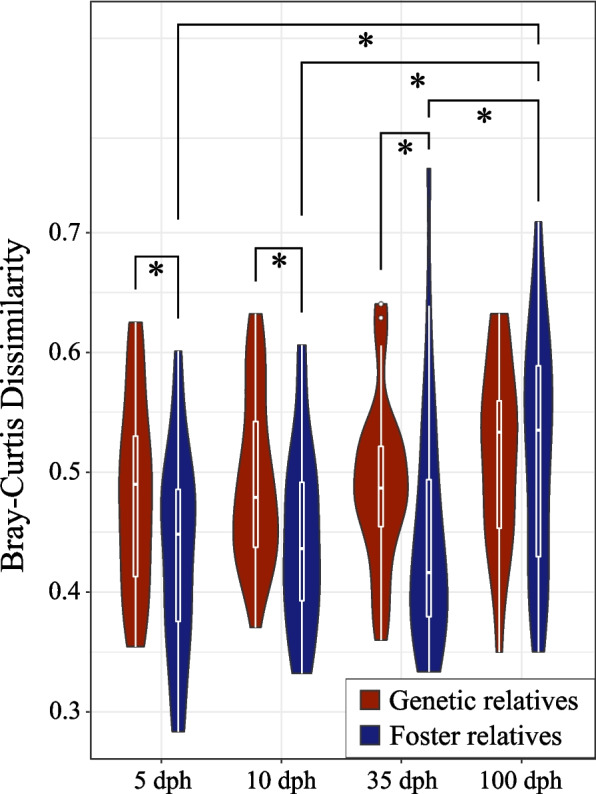
Microbial dissimilarity of zebra finch juveniles reared by unrelated conspecifics with their genetic and foster relatives. Violin plots with embedded box plots comparing the distribution of pairwise BC dissimilarities between zebra finch juveniles reared by unrelated conspecifics and their genetic relatives (genetic mother, father and siblings) and foster relatives (foster mother, father and siblings) at 5, 10 and 35 dph. Significance differences between foster and genetic relatives were determined based on Wilcoxon rank-sum tests. The significance of the change in distance over time in the foster and genetic groups was determined by the Kruskal–Wallis rank-sum test followed by the post hoc Dunn’s test. Significance is highlighted for *p* values ≤ 0.05 (*), *p* ≤ 0.01 (**) and *p* ≤ 0.001 (***) after Bonferroni correction

We analysed the gut microbiota of 12 ZF juveniles reared by unrelated conspecifics. By comparing the dissimilarity between the microbial communities of these juveniles and their genetic and foster relatives, we found that the microbial communities of the juveniles were more similar to those of their foster relatives than to those of their genetic relatives at 5 dph (Wilcoxon rank-sum exact test: *p*=0.04), 10 dph (Wilcoxon rank-sum exact test: *p*=0.017) and 35 dph (Wilcoxon rank-sum exact test: *p*=0.018) (Fig. 8). At 100 dph, there was no significant difference in the similarity of microbial communities between juveniles reared by unrelated conspecifics and their genetic and foster relatives. The distance between the microbial communities of juveniles and those of their foster relatives increased over time (Kruskal–Wallis rank-sum test, *p*=0.011), with significant differences between 5 and 100 dph (post hoc Dunn's test, *p*=0.016), 10 and 100 dph (post hoc Dunn’s test, *p*=0.048) and 35 and 100 dph (post hoc Dunn’s test, *p*=0.041) (Fig. 8). In contrast, the distance between the microbial communities of juveniles and their genetic relatives did not change over time. In the groups comparing juveniles and their mothers and juveniles and their siblings, microbial distances did not significantly differ between genetic and foster relatives, with one exception: at 10 dph, zebra finches had a gut microbial composition more similar to their foster siblings than to their genetic siblings (Kruskal–Wallis rank-sum test, *p*=0.04). In the groups comparing juveniles and their fathers, we did not detect any difference between the genetic and foster groups at any sampling time. However, the distance between the microbial communities of juveniles and their foster fathers increased as they developed (Kruskal–Wallis rank-sum test, *p*=0.033).

Likewise, we analysed the gut microbiota of six BF juveniles reared by unrelated conspecifics. We did not detect any difference in the similarity of microbial communities of juveniles with their genetic relatives and foster relatives at any sampling time. The microbial dissimilarity between juveniles and both their genetic (Kruskal–Wallis rank-sum test, *p*=0.026) and foster relatives (Kruskal–Wallis rank-sum test, *p*=0.004) fluctuated over time, with a significant reduction between 10 and 35 dph in both genetic (post hoc Dunn’s test, *p*=0.01) and foster groups (post hoc Dunn’s test, *p*=0.005). The microbial similarity of juveniles and their siblings did not vary between the genetic and foster groups at any sampling time. Nonetheless, the microbial similarity between BF juveniles and their genetic siblings increased over time (Kruskal–Wallis rank-sum test, *p*=0.03626). The extent of the microbial similarity did not differ between genetic and foster relatives in the groups comparing juvenile and their mothers and juvenile and their fathers.

## Discussion

### Changes in microbial composition over host ontogeny

Microbial communities acquired during early development have been increasingly recognised as an essential component of host fitness due to their impact on host metabolism [90], nervous system development [6, 91] and immune priming [3, 4]. However, a proper understanding of how the microbiota is acquired and how it develops from early life to adulthood is lacking. This study characterised ontogenetic changes in the gut microbiota of zebra finches, a well-studied model organism, and of Bengalese finches over 3 months, a period that covered different developmental stages. The gut microbiota of these species underwent substantial changes with host development. In both species, juveniles exhibited higher microbial diversity than adults at the post-breeding stage, confirming a previous study that showed that zebra finch juveniles had higher alpha diversity of the gut microbiota until 10 dph under controlled conditions [22]. However, studies conducted with natural populations of barn swallows (*Hirundo rustica*) [92], house sparrows (*Passer domesticus*) [93], ostriches (*Struthio camelus*) [53], chinstrap penguins (*Pygoscelis antarctica*) [94] and Eurasian kestrels (*Falco tinnunculus*) [54] reported higher alpha diversity in adults. This inconsistency can be explained by adults being exposed to a much more diverse reservoir of microorganisms and food sources due to their mobility in natural settings. In contrast, nestlings receive food from limited sources, and their most likely source of microorganisms is nest material [22, 95].

In addition to microbial diversity, the taxonomic composition and structure of the microbial communities varied over the course of ontogeny. We found significant compositional changes among juvenile age groups, especially nonconsecutive ones. Furthermore, according to the differential abundance analyses, several microbial taxa became either less prevalent or were recruited throughout the development of both species. Additionally, in both species, most differentially abundant OTUs between the samples collected at 5 and 10 dph were more abundant in the younger age group, indicating loss of initial colonisers. In zebra finches, the most prominent alterations in OTU abundances occurred between 10 and 35 dph, probably due to the transition from parent-dependent feeding to nutritional independence, which occurs at approximately 35 dph [96]. Interestingly, Bengalese finches exhibited the least change in OTU abundances between 10 and 35 dph but the highest between 35 and 100 dph. When comparing adults and 100 dph juveniles, we identified several differentially abundant OTUs in both species, indicating that the microbial communities still underwent substantial changes after 100 dph. Our findings are in line with previous studies showing differences among juvenile age groups [55, 97, 98] as well as juveniles and adults [53, 92, 93, 99].

In summary, these results indicate a gradual progression towards an adult-like microbiota. Nevertheless, the microbial communities of juveniles did not fully converge to an adult state even by 100 dph, indicating that maturation of the gut microbiota takes longer than sexual maturation, which occurs at approximately 90 dph in zebra finches [96]. In altricial birds, juveniles undergo tremendous developmental changes during early life. Given the extent and pace of ontogenetic modifications affecting their gut anatomy, physiology, digestive capacity, metabolism and immune system [51, 52, 100], it is likely that these modifications translate into the changes in the gut microbiota documented in our study.

### The impact of social transmission and host selection on the gut microbiota is age-dependent

Disentangling the relative impacts of host and external factors, particularly those of the rearing environment, has been one of the essential tasks in avian microbial ecology. However, studies have reported inconsistent findings. For example, some investigations have found that the gut microbiota of juvenile birds raised by adults of another species is more similar to that of their conspecifics [43, 46, 47], indicating that host selection due to species-specific characteristics outweighs the impact of the rearing environment. In contrast, other studies have found that the rearing environment has a larger effect on the gut microbiota than host taxonomy [22, 49]. Similarly, the role of the individual genetic background in shaping the gut microbiota is equivocal. In some bird species, genetically related individuals exhibit higher microbial similarity [101–103]. However, in other species, such as great tits (*Parus major*), those raised by unrelated conspecifics have a gut microbiota more similar to their social siblings than to their genetic siblings [50]. A potential explanation for these discrepancies is that most of these studies have been conducted in natural populations where several confounding factors can potentially mask the impact of host factors. Additionally, most of them were conducted during the hatchling or nestling period. However, several key transitions between fledging and maturation can potentially affect the gut microbiota. Thus, our study fills two critical gaps. First, we conducted the experiments under strictly controlled dietary and environmental conditions. This control enabled us to minimise the impact of confounding factors in the rearing environment while manipulating the initial microbial sources, i.e. the social families, by cross-fostering eggs. Therefore, we could directly test whether the gut microbiota of the developing juveniles is a random subset of the parental pool of microorganisms or selective mechanisms prevent the survival of some microbial species occurrence. Second, we collected longitudinal data from the same juveniles over 3 months, covering different developmental stages. Consequently, our study provides a comprehensive picture of the factors involved in the ontogenesis of the gut microbiota, rather than a snapshot of an early developmental phase.

The gut microbiota of heterospecific foster juveniles was more similar to that of their social parents, particularly their foster mothers, during early development. During this period, host selection is weak, and random processes such as dispersal between hosts and from the environment govern the colonisation of the gut [104], making the pool of available microorganisms a crucial determinant of the assembly process. In the early rearing environment of these species, the primary sources of microbial colonisation are nesting material and parental contact [22, 105]. The division of labour between the parents, particularly incubation and food provisioning, can be female-biased even in species with biparental care, such as zebra finches [106]. Consequently, females have more opportunities to exchange microbes with their young. Investigation of the samples collected at 10 and 35 dph revealed a gradual increase in microbial similarity between heterospecific foster juveniles and their genetic relatives, particularly their genetic siblings. Collectively, these results highlight the existence of age-dependent host selection. As hosts grow, they develop selection mechanisms that facilitate the proliferation of microbial taxa best suited to the host’s species-specific requirements.

Our conspecific cross-fostering experiment demonstrated that until 35 dph, the microbial communities of zebra finch juveniles resembled those of their nestmates. However, at 100 dph, they were less similar to their social group members, indicating that host genetic background becomes more critical in shaping the gut microbiota as zebra finches mature. These findings are in line with previous studies on shrimp [59, 107] and fish [2, 56–58], which showed that the early-life gut microbiota resembles the rearing environment, with similarity decreasing as the hosts develop. Although increased similarity with genetic relatives was evident only for sibling groups in Bengalese finches, it is important to note that this juvenile group had a relatively small sample size (*N*=6).

Increases in host selection with maturation can be explained by age-specific alterations in the gastrointestinal habitat. The avian gut undergoes dramatic modifications during early development, affecting anatomical, physiological, and metabolic conditions [100]. Therefore, it would be reasonable to assume that species-specific and individual differences become more prominent after these developmental changes occur, making selection by the gut habitat a more influential constraint on microbial proliferation. However, these assumptions warrant further investigation. Another nonexclusive explanation is that nestlings do not have a fully developed immune system capable of selecting for specific microorganisms during early development. Supporting this hypothesis, some components of adaptive and innate immunity are immature in nestlings of several bird species, even close to fledging [52, 108–110]. For example, adaptive immunity is not fully mature in zebra finches at least until 21 dph [51]. Similarly, in chickens, the development of gut-associated lymphoid tissue that provides mucosal immunity is not complete until 16 weeks after hatching [111]. Species-specific and individual genetic differences in immune-related genes are likely to increase in importance for sculpting the gut microbiota after the maturation of the immune system. However, the exact links between the developing immune system and host selection have yet to be determined. Notably, host selection was evident in the heterospecific cross-fostering experiments starting from 10 dph, while it occurred only at 100 dph in the conspecific cross-fostering experiment. This difference in timing indicates that species-specific characteristics are more powerful determinants of host selection than the individual genetic background.

## Conclusion

Overall, our study documented developmental changes in the diversity, composition and structure of the gut microbiota of zebra finches and Bengalese finches, providing a baseline description for further study of the impact of the early gut microbiota on host fitness. Our study provides one of the first and most comprehensive analyses of how the social environment and host selection interact to shape the assembly and ontogenesis of the gut microbiota. We demonstrated that in the early stages of life, the gut microbiota largely resembles the microbial reservoir of nestmates. In later stages, the social environment is still influential, despite the increasing impact of host selection. Thus, we demonstrated that the timing of key transitions, such as gut maturation and the development of the immune system, should be considered when investigating determinants of the gut microbiota. This essential but often neglected point in avian microbiome studies is also important for the experimental manipulation of early microbial colonies. These pioneering findings broaden our understanding of the ecological processes governing the assembly and ontogenesis of the host-associated microbiota in birds, suggesting new avenues to study the mechanisms of host selection.

## Supplementary Information

**Additional file 1.** Sample numbers used in the study.

**Additional file 2.** LMM investigating alpha diversity in zebra finches across ontogenetic stages. P-values ≤ 0.05 are shown in bold.

**Additional file 3.** LMM investigating alpha diversity in Bengalese finches across ontogenetic stages. P-values ≤ 0.05 are shown in bold.

**Additional file 4.** nMDS plots of the dissimilarities of the gut microbiota across Zebra finch Ontogeny based on (A) Bray-Curtis dissimilarities, (B) Weighted UniFrac distances.

**Additional file 5.** nMDS plots of the dissimilarities of the gut microbiota across Bengalese finch Ontogeny based on (A) Bray-Curtis dissimilarities, (B) Weighted UniFrac distances.

**Additional file 6.** The mean abundance and the standard deviation of the microbial families identified in the zebra finches.

**Additional file 7.** The mean abundance and the standard deviation of the microbial families identified in the Bengalese finch.

**Additional file 8.** LMM investigatigating alpha diversity in different sample types across time.

**Additional file 9.** Alpha diversity of different sample types based on (A) Shannon's diversity index, and (B) Faith's phylogenetic diversity at 5, 10 and 35 dph. The lines within the box plots indicate the medians, and the lower and upper boundaries of the boxes indicate the 25th and 75th percentiles, respectively. Whiskers above and below the boxes correspond to 1.5 times the interquartile range (IQR) above and below the 25th and 75th percentiles, respectively.

**Additional file 10.** Indicator OTUs for sample types at each sampling time. IndVal index is calculated as the product of specificity (A) and fidelity (B) values ranging between 0 and 1 (1 indicates the taxon exclusively occurs in the given group).

## Data Availability

The datasets generated during the current study can be found in the European Nucleotide Archive repository, Project ID: PRJEB53212. The code used in the analyses is available in the GitHub repository at https://github.com/AnnaAntonatouPap/Timming-matters.

## Abbreviations

BC: Bray–Curtis
BF: Bengalese finch
dph: Days post-hatch
LMM: Linear mixed models
nMDS: Nonmetric multidimensional scaling
OTU: Operational taxonomic unit
PERMANOVA: Permutational multivariate analysis of variance
rRNA: Ribosomal RNA
WU: Weighted UniFrac
ZF: Zebra finch
