# Supplementary material for: Timing matters: age-dependent impacts of the social environment and host selection on the avian gut microbiota
Source: Microbiome. 2022 Nov 26;10:202. doi: 10.1186/s40168-022-01401-0 (PMC9700942; doi:10.1186/s40168-022-01401-0)
Supplement: Supplementary file 2 — Additional file 1. Sample numbers used in the study. [file 40168_2022_1401_MOESM1_ESM.pdf]

Additional file 1. Sample numbers used in the study.

Sample types		N Individuals	N Samples	
			Initial Dataset	Final dataset
ZF adults	ZF Females	14	56	56
	ZF Males	14	56	54
BF adults	BF Females	7*	36	31
	BF Males	7*	36	32
BF juveniles reared by BF adults	BF reared by genetic parents	11	44	44
	BF reared by unrelated conspecifics	6	24	23
ZF juveniles reared by ZF adults	ZF reared by genetic parents	17	68	63
	ZF reared by unrelated conspecifics	11	44	40
ZF juveniles reared by BF adults	ZF Reared by BF adults	8	32	32
Total		95	396	375

*2 birds were used in two different, successive breeding events, each assigned to a different fostering experiment.
