# Supplementary material for: Timing matters: age-dependent impacts of the social environment and host selection on the avian gut microbiota
Source: Microbiome. 2022 Nov 26;10:202. doi: 10.1186/s40168-022-01401-0 (PMC9700942; doi:10.1186/s40168-022-01401-0)
Supplement: Supplementary file 3 — Additional file 2. LMM investigating alpha diversity in zebra finches across ontogenetic stages. P-values ≤ 0.05 are shown in bold. [file 40168_2022_1401_MOESM2_ESM.pdf]

Additional file 2. LMM investigating alpha diversity in zebra finches across ontogenetic stages. P-values ≤ 0.05 are shown in bold.

Zebra Finch Shannon's Diversity Index					
**LMM**	**Est**	**SE**	**CI**		**p**
Intercept	0.96	0.09	0.77	1.15	<0.001
**Random Effects**					
δ2		0.17			
τ00 (Rearing nest)		0.03			
ICC		0.15			
N (Rearing nest)		14			
Observations		129			
Marginal R2		0.135			
Conditional R2		0.263			

Pairwise Comparisons

**Group 1**	**Group 2**	**Est**	**SE**	**t**	**p**
ZF juvenile Day5	ZF adults	0.453	0.116	3.911	**<0.001**
ZF juvenile Day10	ZF adults	0.134	0.114	1.171	0.244
ZF juvenile Day35	ZF adults	0.424	0.116	3.658	**<0.001**
ZF juvenile Day100	ZF adults	0.333	0.118	2.811	**0.006**
ZF juvenile Day10	ZF juvenile Day5	0.319	0.112	2.863	**0.005**
ZF juvenile Day35	ZF juvenile Day5	0.029	0.113	0.258	0.797
ZF juvenile Day100	ZF juvenile Day5	0.120	0.116	1.033	0.303
ZF juvenile Day35	ZF juvenile Day10	0.290	0.112	2.600	**0.01**
ZF juvenile Day100	ZF juvenile Day10	0.199	0.114	1.746	0.083
ZF juvenile Day100	ZF juvenile Day35	0.091	0.116	0.781	0.436

Zebra Finch Faith's Phylogenetic Diversity Index					
**LMM**	**Est.**	**SE**	**CI**		**p**
Intercept	2.97	0.09	2.79	3.14	<0.001
**Random Effects**					
δ2	0.19				
τ00 (Rearing nest)	0.01				
ICC	0.04				
N (Rearing nest)	14				
Observations	129				
Marginal R2	0.068				
Conditional R2	0.105				

Pairwise Comparisons

**Group 1**	**Group 2**	**Est**	**SE**	**t value**	**p**
ZF juvenile Day5	ZF adults	0.326	0.121	2.684	**0.008**
ZF juvenile Day10	ZF adults	0.252	0.119	2.111	**0.037**
ZF juvenile Day35	ZF adults	0.327	0.121	2.688	**0.008**
ZF juvenile Day100	ZF adults	0.251	0.124	2.022	**0.045**
ZF juvenile Day10	ZF juvenile Day5	0.074	0.118	0.628	0.531
ZF juvenile Day35	ZF juvenile Day5	0.001	0.120	0.004	0.996
ZF juvenile Day100	ZF juvenile Day5	0.075	0.122	0.614	0.54
ZF juvenile Day35	ZF juvenile Day10	0.074	0.118	0.632	0.528
ZF juvenile Day100	ZF juvenile Day10	0.001	0.120	0.011	0.991
ZF juvenile Day100	ZF juvenile Day35	0.076	0.122	0.618	0.537
