# Supplementary material for: Timing matters: age-dependent impacts of the social environment and host selection on the avian gut microbiota
Source: Microbiome. 2022 Nov 26;10:202. doi: 10.1186/s40168-022-01401-0 (PMC9700942; doi:10.1186/s40168-022-01401-0)
Supplement: Supplementary file 4 — Additional file 3. LMM investigating alpha diversity in Bengalese finches across ontogenetic stages. P-values ≤ 0.05 are shown in bold. [file 40168_2022_1401_MOESM3_ESM.pdf]

Additional file 3. LMM investigating alpha diversity in Bengalese finches across ontogenetic stages. P-values ≤ 0.05 are shown in bold.

Bengalese finch Shannon's Diversity						
**LMM**		**Est.**	**Std. Er.**	**CI**		**p**
Intercept		0.94	14	0.66	1.22	<0.001
**Random Effects**						
δ2	0.2					
τ00 (Rearing nest)	0.01					
ICC	0.04					
N (Rearing nest)	8					
Observations	78					
Marginal /Conditional R2	0.045/0.087					
**Pairwise Comparisons**						
**Group 1**	**Group 2**	**Est.**	**Std. Er**	**t**		**p**
BF juvenile Day5	BF adult Day100	0.151	0.176	0.855		0.395
BF juvenile Day10	BF adult Day100	0.098	0.174	0.561		0.576
BF juvenile Day35	BF adult Day100	0.300	0.174	1.726		0.088
BF juvenile Day100	BF adult Day100	0.232	0.174	1.333		0.186
BF juvenile Day10	BF juvenile Day5	0.053	0.156	0.341		0.734
BF juvenile Day35	BF juvenile Day5	0.149	0.156	0.958		0.341
BF juvenile Day100	BF juvenile Day5	0.081	0.156	0.520		0.604
BF juvenile Day35	BF juvenile Day10	0.203	0.153	1.320		0.191
BF juvenile Day100	BF juvenile Day10	0.134	0.153	0.875		0.384
BF juvenile Day100	BF juvenile Day35	0.068	0.153	0.445		0.658

Bengalese finch Faith's PD						
**LMM**		**Est.**	**Std. Er.**	**CI**		**p**
Intercept		2.86	0.14	2.59	3.14	<0.001
**Random Effects**						
δ2	0.18					
τ00 (Rearing nest)	0.02					
ICC	0.1					
N (Rearing nest)	8					
Observations	78					
Marginal/Conditional R2	0.129/0.217					
**Pairwise Comparisons**						
**Group 1**	**Group 2**	**Est.**	**Std. Er.**	**t**		**p**
BF juvenile Day5	BF adult Day100	0.579	0.16676	3.472		**<0.001**
BF juvenile Day10	BF adult Day100	0.34388	0.16443	2.091		**0.04**
BF juvenile Day35	BF adult Day100	0.41201	0.16443	2.506		**0.014**
BF juvenile Day100	BF adult Day100	0.32722	0.16443	1.99		**0.050**
BF juvenile Day10	BF juvenile Day5	0.23513	0.14698	1.6		0.114
BF juvenile Day35	BF juvenile Day5	0.16699	0.14698	1.136		0.259
BF juvenile Day100	BF juvenile Day5	0.25178	0.14698	1.713		0.091
BF juvenile Day35	BF juvenile Day10	0.06814	0.14462	0.471		0.639
BF juvenile Day100	BF juvenile Day10	0.01665	0.14462	0.115		0.909
BF juvenile Day100	BF juvenile Day35	0.08479	0.14462	0.586		0.559
