# Supplementary material for: Timing matters: age-dependent impacts of the social environment and host selection on the avian gut microbiota
Source: Microbiome. 2022 Nov 26;10:202. doi: 10.1186/s40168-022-01401-0 (PMC9700942; doi:10.1186/s40168-022-01401-0)

Additional file 5. nMDS plots of the dissimilarities of the gut microbiota across Bengalese finch Ontogeny based on (A) Bray-Curtis dissimilarities, (B) Weighted UniFrac distances.

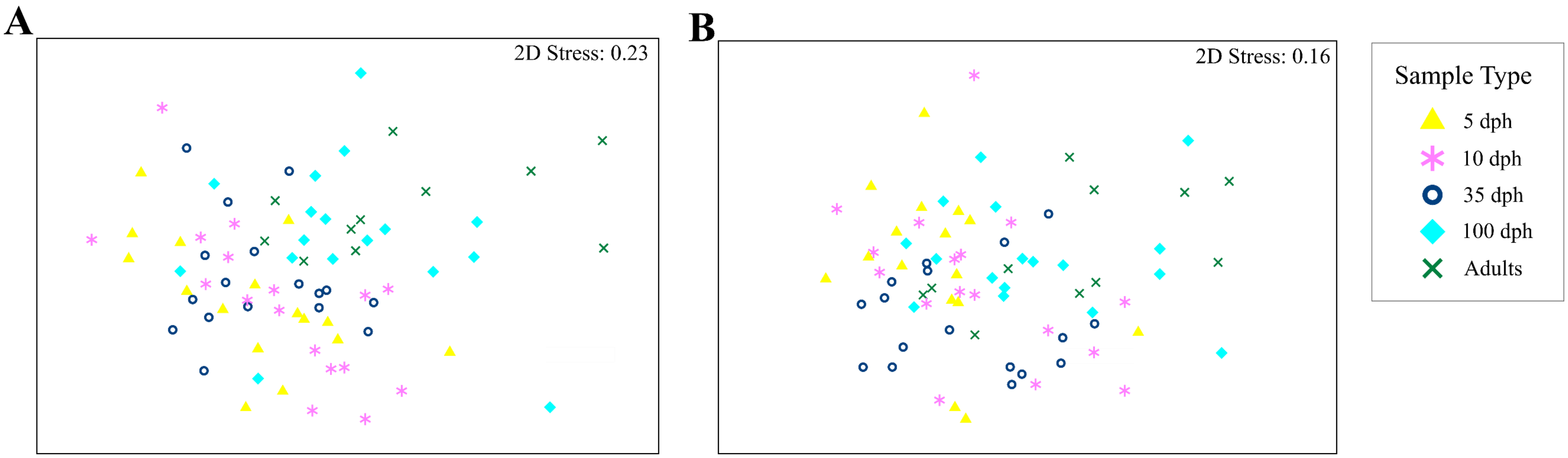

Supplement: Supplementary file 6 — Additional file 5. nMDS plots of the dissimilarities of the gut microbiota across Bengalese finch Ontogeny based on (A) Bray-Curtis dissimilarities, (B) Weighted UniFrac distances. [file 40168_2022_1401_MOESM5_ESM.pdf]
