# Supplementary material for: Timing matters: age-dependent impacts of the social environment and host selection on the avian gut microbiota
Source: Microbiome. 2022 Nov 26;10:202. doi: 10.1186/s40168-022-01401-0 (PMC9700942; doi:10.1186/s40168-022-01401-0)
Supplement: Supplementary file 8 — Additional file 7. The mean abundance and the standard deviation of the microbial families identified in the Bengalese finch. [file 40168_2022_1401_MOESM7_ESM.pdf]

Additional file 7. The mean abundance and the standard deviation of the microbial families identified in the Bengalese finch.

Microbial Family	5-dph		10-dph		35-dph		100-dph		Adults	
	Mean	SD	Mean	SD	Mean	SD	Mean	SD	Mean	SD
Campylobacteraceae	0.4404	0.3482	0.6022	0.2369	0.3898	0.1880	0.2375	0.1886	0.1350	0.0939
Lactobacillaceae	0.3708	0.3222	0.3206	0.2048	0.5263	0.1950	0.6669	0.2036	0.8291	0.0907
Enterobacteriaceae	0.0596	0.1339	0.0076	0.0164	0.0197	0.0186	0.0232	0.0652	0.0083	0.0233
Leuconostocaceae	0.0412	0.0789	0.0089	0.0087	0.0190	0.0279	0.0033	0.0069	0.0016	0.0022
Bifidobacteriaceae	0.0231	0.0321	0.0152	0.0194	0.0058	0.0071	0.0082	0.0118	0.0049	0.0128
Veillonellaceae	0.0169	0.0308	0.0101	0.0175	0.0049	0.0080	0.0058	0.0073	0.0040	0.0077
Enterococcaceae	0.0118	0.0237	0.0093	0.0146	0.0145	0.0152	0.0109	0.0137	0.0023	0.0042
Pseudomonadaceae	0.0071	0.0211	0.0010	0.0028	0.0006	0.0014	0.0008	0.0022	0.0007	0.0022
Helicobacteraceae	0.0047	0.0128	0.0138	0.0359	0.0098	0.0122	0.0137	0.0198	0.0074	0.0070
Micrococcaceae	0.0042	0.0075	0.0014	0.0014	0.0019	0.0032	0.0089	0.0116	0.0029	0.0024
Erwiniaceae	0.0040	0.0091	0.0022	0.0072	0.0026	0.0051	0.0014	0.0028	0.0005	0.0014
Streptococcaceae	0.0039	0.0066	0.0011	0.0011	0.0009	0.0009	0.0014	0.0014	0.0005	0.0004
Moraxellaceae	0.0025	0.0055	0.0014	0.0038	0.0002	0.0002	0.0003	0.0004	0.0002	0.0004
Atopobiaceae	0.0020	0.0053	0.0024	0.0082	0.0002	0.0002	0.0046	0.0137	0.0004	0.0011
Pasteurellaceae	0.0013	0.0038	0.0001	0.0001	0.0002	0.0003	0.0066	0.0184	0.0002	0.0003
Bacillaceae	0.0012	0.0022	0.0003	0.0006	0.0001	0.0002	0.0000	0.0000	0.0000	0.0000
Micrococcales_unclassified	0.0010	0.0025	0.0003	0.0008	0.0002	0.0002	0.0011	0.0036	0.0001	0.0001
Staphylococcaceae	0.0004	0.0004	0.0002	0.0005	0.0001	0.0001	0.0002	0.0002	0.0000	0.0000
Corynebacteriaceae	0.0003	0.0004	0.0002	0.0002	0.0005	0.0005	0.0001	0.0001	0.0002	0.0003
Microbacteriaceae	0.0003	0.0008	0.0001	0.0001	0.0001	0.0001	0.0001	0.0001	0.0001	0.0001
Rhizobiaceae	0.0003	0.0004	0.0001	0.0001	0.0001	0.0001	0.0000	0.0000	0.0000	0.0000
Rhodobacteraceae	0.0003	0.0007	0.0003	0.0007	0.0000	0.0001	0.0001	0.0001	0.0003	0.0003
Campylobacterales_unclassified	0.0003	0.0011	0.0001	0.0002	0.0002	0.0005	0.0000	0.0000	0.0000	0.0000
Yersiniaceae	0.0002	0.0009	0.0000	0.0000	0.0000	0.0000	0.0000	0.0000	0.0000	0.0000
Enterobacterales_unclassified	0.0002	0.0003	0.0000	0.0001	0.0002	0.0002	0.0001	0.0002	0.0001	0.0001
Paenibacillaceae	0.0002	0.0006	0.0000	0.0001	0.0001	0.0001	0.0000	0.0000	0.0000	0.0000
Alcaligenaceae	0.0001	0.0003	0.0001	0.0001	0.0001	0.0002	0.0000	0.0001	0.0000	0.0000
Comamonadaceae	0.0001	0.0002	0.0001	0.0001	0.0000	0.0000	0.0000	0.0001	0.0000	0.0000
Propionibacteriaceae	0.0001	0.0002	0.0000	0.0001	0.0000	0.0000	0.0000	0.0000	0.0000	0.0000
Actinobacteria_unclassified	0.0001	0.0002	0.0000	0.0001	0.0000	0.0001	0.0000	0.0000	0.0001	0.0001
Xanthomonadaceae	0.0001	0.0002	0.0000	0.0001	0.0000	0.0000	0.0000	0.0000	0.0000	0.0000
Flavobacteriaceae	0.0001	0.0003	0.0000	0.0000	0.0000	0.0000	0.0000	0.0000	0.0000	0.0000
Sphingomonadaceae	0.0001	0.0001	0.0000	0.0001	0.0000	0.0000	0.0000	0.0000	0.0000	0.0000
Diplorickettsiaceae	0.0001	0.0002	0.0000	0.0001	0.0000	0.0000	0.0000	0.0000	0.0000	0.0000
Beijerinckiaceae	0.0001	0.0001	0.0000	0.0000	0.0000	0.0000	0.0000	0.0000	0.0000	0.0000
Clostridiaceae	0.0001	0.0002	0.0001	0.0002	0.0001	0.0002	0.0003	0.0013	0.0000	0.0000
Caulobacteraceae	0.0001	0.0001	0.0000	0.0000	0.0000	0.0000	0.0000	0.0000	0.0000	0.0000
Planococcaceae	0.0000	0.0001	0.0002	0.0005	0.0000	0.0000	0.0000	0.0000	0.0000	0.0000
Xanthobacteraceae	0.0000	0.0001	0.0000	0.0000	0.0000	0.0000	0.0000	0.0000	0.0000	0.0000
Chloroflexaceae	0.0000	0.0002	0.0000	0.0000	0.0000	0.0000	0.0000	0.0000	0.0000	0.0000
Neisseriaceae	0.0000	0.0001	0.0000	0.0001	0.0000	0.0000	0.0000	0.0000	0.0000	0.0000
Sphingobacteriaceae	0.0000	0.0001	0.0000	0.0000	0.0000	0.0000	0.0000	0.0000	0.0000	0.0000
Lactobacillales_unclassified	0.0000	0.0001	0.0001	0.0004	0.0005	0.0015	0.0001	0.0001	0.0002	0.0002
Nostocaceae	0.0000	0.0001	0.0000	0.0000	0.0000	0.0000	0.0000	0.0000	0.0000	0.0000
Pyrinomonadaceae	0.0000	0.0001	0.0000	0.0000	0.0000	0.0000	0.0000	0.0000	0.0000	0.0000
Aeromonadaceae	0.0000	0.0001	0.0000	0.0000	0.0000	0.0000	0.0000	0.0000	0.0000	0.0000

Coleofasciculaceae	0.0000	0.0001	0.0000	0.0000	0.0000	0.0000	0.0000	0.0000	0.0000	0.0000
Actinomycetaceae	0.0000	0.0001	0.0000	0.0000	0.0000	0.0000	0.0000	0.0000	0.0000	0.0000
Dermabacteraceae	0.0000	0.0001	0.0000	0.0001	0.0000	0.0000	0.0000	0.0000	0.0000	0.0000
Carnobacteriaceae	0.0000	0.0001	0.0000	0.0001	0.0000	0.0000	0.0000	0.0000	0.0000	0.0001
Gammaproteobacteria_unclassified	0.0000	0.0000	0.0000	0.0000	0.0000	0.0000	0.0000	0.0000	0.0000	0.0000
Devosiaceae	0.0000	0.0001	0.0000	0.0000	0.0000	0.0000	0.0000	0.0000	0.0000	0.0000
Nocardiaceae	0.0000	0.0001	0.0000	0.0000	0.0000	0.0000	0.0000	0.0000	0.0000	0.0000
Sanguibacteraceae	0.0000	0.0001	0.0000	0.0001	0.0000	0.0000	0.0000	0.0000	0.0000	0.0000
Catenulisporaceae	0.0000	0.0001	0.0000	0.0000	0.0000	0.0000	0.0000	0.0000	0.0000	0.0000
Halomonadaceae	0.0000	0.0000	0.0000	0.0000	0.0000	0.0000	0.0000	0.0000	0.0000	0.0000
Blastocatellaceae	0.0000	0.0000	0.0000	0.0000	0.0000	0.0000	0.0000	0.0000	0.0000	0.0000
Ruminococcaceae	0.0000	0.0000	0.0000	0.0000	0.0000	0.0000	0.0000	0.0000	0.0000	0.0000
Weeksellaceae	0.0000	0.0000	0.0000	0.0000	0.0000	0.0000	0.0000	0.0000	0.0000	0.0000
Leptotrichiaceae	0.0000	0.0000	0.0000	0.0000	0.0000	0.0000	0.0000	0.0000	0.0000	0.0000
A4b	0.0000	0.0000	0.0000	0.0000	0.0000	0.0000	0.0000	0.0000	0.0000	0.0000
Oxalobacteraceae	0.0000	0.0000	0.0000	0.0000	0.0000	0.0000	0.0000	0.0000	0.0000	0.0000
Solirubrobacteraceae	0.0000	0.0000	0.0000	0.0000	0.0000	0.0000	0.0000	0.0000	0.0000	0.0000
Nocardioidaceae	0.0000	0.0000	0.0000	0.0000	0.0000	0.0000	0.0000	0.0000	0.0000	0.0000
Deinococcaceae	0.0000	0.0000	0.0000	0.0000	0.0000	0.0000	0.0000	0.0000	0.0000	0.0000
Cyanobacteriales_unclassified	0.0000	0.0000	0.0000	0.0000	0.0000	0.0000	0.0000	0.0000	0.0000	0.0000
Phycisphaerae_unclassified	0.0000	0.0000	0.0000	0.0000	0.0000	0.0000	0.0000	0.0000	0.0000	0.0000
Fusobacteriaceae	0.0000	0.0000	0.0000	0.0000	0.0000	0.0000	0.0000	0.0000	0.0000	0.0000
Mycobacteriaceae	0.0000	0.0000	0.0000	0.0000	0.0000	0.0000	0.0000	0.0000	0.0000	0.0000
Saccharimonadaceae	0.0000	0.0000	0.0000	0.0000	0.0000	0.0000	0.0000	0.0000	0.0000	0.0000
Sericytochromatia_fa	0.0000	0.0000	0.0000	0.0000	0.0000	0.0000	0.0000	0.0000	0.0000	0.0000
Acetobacteraceae	0.0000	0.0000	0.0000	0.0000	0.0000	0.0000	0.0000	0.0000	0.0000	0.0000
Aerococcaceae	0.0000	0.0000	0.0000	0.0000	0.0000	0.0000	0.0000	0.0000	0.0000	0.0000
Firmicutes_unclassified	0.0000	0.0000	0.0000	0.0000	0.0000	0.0000	0.0000	0.0000	0.0000	0.0000
Ilumatobacteraceae	0.0000	0.0000	0.0000	0.0000	0.0000	0.0000	0.0000	0.0000	0.0000	0.0000
Dietziaceae	0.0000	0.0000	0.0000	0.0000	0.0000	0.0000	0.0000	0.0000	0.0000	0.0000
Lachnospiraceae	0.0000	0.0000	0.0000	0.0000	0.0000	0.0000	0.0000	0.0000	0.0000	0.0000
Cyanobiaceae	0.0000	0.0000	0.0000	0.0000	0.0000	0.0000	0.0000	0.0000	0.0000	0.0000
Brachyspiraceae	0.0000	0.0000	0.0003	0.0011	0.0012	0.0035	0.0025	0.0053	0.0010	0.0018
Bacilli_unclassified	0.0000	0.0000	0.0000	0.0000	0.0000	0.0000	0.0000	0.0000	0.0000	0.0000
Catellicoccaceae	0.0000	0.0000	0.0000	0.0000	0.0000	0.0000	0.0017	0.0068	0.0000	0.0000
Spirosomaceae	0.0000	0.0000	0.0000	0.0000	0.0000	0.0000	0.0000	0.0000	0.0000	0.0000
Pseudonocardiaceae	0.0000	0.0000	0.0000	0.0000	0.0000	0.0000	0.0000	0.0002	0.0000	0.0000
Exiguobacteraceae	0.0000	0.0000	0.0000	0.0000	0.0000	0.0000	0.0000	0.0000	0.0000	0.0000
Streptomycetaceae	0.0000	0.0000	0.0000	0.0000	0.0000	0.0000	0.0000	0.0000	0.0000	0.0000
Bacillales_unclassified	0.0000	0.0000	0.0000	0.0001	0.0000	0.0000	0.0000	0.0000	0.0000	0.0000
Corynebacteriales_unclassified	0.0000	0.0000	0.0000	0.0000	0.0000	0.0000	0.0000	0.0000	0.0000	0.0000
uncultured	0.0000	0.0000	0.0000	0.0000	0.0000	0.0000	0.0000	0.0000	0.0000	0.0000
Intrasporangiaceae	0.0000	0.0000	0.0000	0.0000	0.0000	0.0000	0.0000	0.0000	0.0000	0.0000
Thermoactinomycetaceae	0.0000	0.0000	0.0000	0.0000	0.0000	0.0000	0.0000	0.0000	0.0000	0.0000
Nocardiopsaceae	0.0000	0.0000	0.0000	0.0000	0.0000	0.0000	0.0000	0.0000	0.0000	0.0000
Chthoniobacteraceae	0.0000	0.0000	0.0000	0.0000	0.0000	0.0000	0.0000	0.0000	0.0000	0.0000
Azospirillaceae	0.0000	0.0000	0.0000	0.0000	0.0000	0.0000	0.0000	0.0000	0.0000	0.0000
Micromonosporaceae	0.0000	0.0000	0.0000	0.0000	0.0000	0.0000	0.0000	0.0000	0.0000	0.0000
Veillonellales-Selenomonadales_unclassified	0.0000	0.0000	0.0000	0.0000	0.0000	0.0001	0.0000	0.0000	0.0000	0.0000
Brevibacillaceae	0.0000	0.0000	0.0000	0.0000	0.0000	0.0000	0.0000	0.0000	0.0000	0.0000

Rubrobacteriaceae	0.0000	0.0000	0.0000	0.0000	0.0000	0.0000	0.0000	0.0000	0.0000	0.0000
Armatimonadales_fa	0.0000	0.0000	0.0000	0.0001	0.0000	0.0000	0.0000	0.0000	0.0000	0.0000
Peptostreptococcales-Tissierellales_fa	0.0000	0.0000	0.0000	0.0000	0.0000	0.0000	0.0000	0.0000	0.0000	0.0000
Dermacoccaceae	0.0000	0.0000	0.0000	0.0000	0.0000	0.0000	0.0000	0.0000	0.0000	0.0000
Cardiobacteriaceae	0.0000	0.0000	0.0000	0.0000	0.0000	0.0000	0.0000	0.0000	0.0000	0.0000
Acidobacteriaceae_(Subgroup_1)	0.0000	0.0000	0.0000	0.0000	0.0000	0.0000	0.0000	0.0000	0.0000	0.0000
RBG-16-55-12_fa	0.0000	0.0000	0.0000	0.0000	0.0000	0.0000	0.0000	0.0000	0.0000	0.0000
Hymenobacteraceae	0.0000	0.0000	0.0000	0.0000	0.0000	0.0000	0.0000	0.0000	0.0000	0.0000
Chitinophagaceae	0.0000	0.0000	0.0000	0.0000	0.0000	0.0000	0.0000	0.0000	0.0000	0.0000
Mycoplasmataceae	0.0000	0.0000	0.0000	0.0000	0.0000	0.0000	0.0000	0.0000	0.0000	0.0000
Rubritaleaceae	0.0000	0.0000	0.0000	0.0000	0.0000	0.0000	0.0000	0.0000	0.0000	0.0000
Prevotellaceae	0.0000	0.0000	0.0000	0.0000	0.0000	0.0000	0.0000	0.0000	0.0000	0.0000
Parachlamydiaceae	0.0000	0.0000	0.0000	0.0000	0.0000	0.0000	0.0000	0.0000	0.0000	0.0000
Chroococcidiopsaceae	0.0000	0.0000	0.0000	0.0000	0.0000	0.0000	0.0000	0.0000	0.0000	0.0000
Microcystaceae	0.0000	0.0000	0.0000	0.0000	0.0000	0.0000	0.0000	0.0000	0.0000	0.0000
Geodermatophilaceae	0.0000	0.0000	0.0000	0.0000	0.0000	0.0000	0.0000	0.0000	0.0000	0.0000
Symbiobacteraceae	0.0000	0.0000	0.0000	0.0000	0.0000	0.0000	0.0000	0.0000	0.0000	0.0000
Cyanobacteriia_unclassified	0.0000	0.0000	0.0000	0.0000	0.0000	0.0000	0.0000	0.0000	0.0000	0.0000
uncultured	0.0000	0.0000	0.0000	0.0000	0.0000	0.0000	0.0000	0.0000	0.0000	0.0000
AKIW781	0.0000	0.0000	0.0000	0.0000	0.0000	0.0000	0.0000	0.0000	0.0000	0.0000
Erysipelotrichaceae	0.0000	0.0000	0.0000	0.0000	0.0000	0.0000	0.0000	0.0000	0.0000	0.0000
Gemmatimonadaceae	0.0000	0.0000	0.0000	0.0000	0.0000	0.0000	0.0000	0.0000	0.0000	0.0000
uncultured	0.0000	0.0000	0.0000	0.0000	0.0000	0.0000	0.0000	0.0000	0.0000	0.0000
Porphyromonadaceae	0.0000	0.0000	0.0000	0.0000	0.0000	0.0000	0.0000	0.0000	0.0000	0.0000
Ktedonobacteraceae	0.0000	0.0000	0.0000	0.0000	0.0000	0.0000	0.0000	0.0000	0.0000	0.0000
Pirellulaceae	0.0000	0.0000	0.0000	0.0000	0.0000	0.0000	0.0000	0.0000	0.0000	0.0000
uncultured	0.0000	0.0000	0.0000	0.0000	0.0000	0.0000	0.0000	0.0000	0.0000	0.0000
Rhizobiales_Incertae_Sedis	0.0000	0.0000	0.0000	0.0000	0.0000	0.0000	0.0000	0.0000	0.0000	0.0000
WD2101_soil_group	0.0000	0.0000	0.0000	0.0000	0.0000	0.0000	0.0000	0.0000	0.0000	0.0000
Oscillospiraceae	0.0000	0.0000	0.0000	0.0000	0.0000	0.0000	0.0000	0.0000	0.0000	0.0000
Opitutaceae	0.0000	0.0000	0.0000	0.0000	0.0000	0.0000	0.0000	0.0000	0.0000	0.0000
Paludibacteraceae	0.0000	0.0000	0.0000	0.0000	0.0000	0.0000	0.0000	0.0000	0.0000	0.0000
Dysgonomonadaceae	0.0000	0.0000	0.0000	0.0000	0.0000	0.0000	0.0000	0.0000	0.0000	0.0000
Ethanoligenenaceae	0.0000	0.0000	0.0000	0.0000	0.0000	0.0000	0.0000	0.0000	0.0000	0.0000
Saccharimonadales_unclassified	0.0000	0.0000	0.0000	0.0000	0.0000	0.0000	0.0000	0.0000	0.0000	0.0000
Kineosporiaceae	0.0000	0.0000	0.0000	0.0000	0.0000	0.0000	0.0000	0.0000	0.0000	0.0000
Burkholderiaceae	0.0000	0.0000	0.0000	0.0000	0.0000	0.0000	0.0000	0.0000	0.0000	0.0000
1-20_fa	0.0000	0.0000	0.0000	0.0000	0.0000	0.0000	0.0000	0.0000	0.0000	0.0000
37-13	0.0000	0.0000	0.0000	0.0000	0.0000	0.0000	0.0000	0.0000	0.0000	0.0000
67-14	0.0000	0.0000	0.0000	0.0000	0.0000	0.0000	0.0000	0.0000	0.0000	0.0000
Abditibacteriaceae	0.0000	0.0000	0.0000	0.0000	0.0000	0.0000	0.0000	0.0000	0.0000	0.0000
Acidaminococcaceae	0.0000	0.0000	0.0000	0.0000	0.0000	0.0000	0.0000	0.0000	0.0000	0.0000
Actinobacteriota_unclassified	0.0000	0.0000	0.0000	0.0000	0.0000	0.0000	0.0000	0.0000	0.0000	0.0000
Actinomarinaceae	0.0000	0.0000	0.0000	0.0000	0.0000	0.0000	0.0000	0.0000	0.0000	0.0000
AEGEAN-169_marine_group	0.0000	0.0000	0.0000	0.0000	0.0000	0.0000	0.0000	0.0000	0.0000	0.0000
Alicyclobacillaceae	0.0000	0.0000	0.0000	0.0000	0.0000	0.0000	0.0000	0.0000	0.0000	0.0000
Alphaproteobacteria_unclassified	0.0000	0.0000	0.0000	0.0000	0.0000	0.0000	0.0000	0.0000	0.0000	0.0000
Alteromonadaceae	0.0000	0.0000	0.0000	0.0000	0.0000	0.0000	0.0000	0.0000	0.0000	0.0000
Anaerolineaceae	0.0000	0.0000	0.0000	0.0000	0.0000	0.0000	0.0000	0.0000	0.0000	0.0000
Arcobacteraceae	0.0000	0.0000	0.0000	0.0000	0.0000	0.0000	0.0000	0.0000	0.0000	0.0000

Arenicellaceae	0.0000	0.0000	0.0000	0.0000	0.0000	0.0000	0.0000	0.0000	0.0000	0.0000
Bacteriovoracaceae	0.0000	0.0000	0.0000	0.0000	0.0000	0.0000	0.0000	0.0000	0.0000	0.0000
Brevibacteriaceae	0.0000	0.0000	0.0000	0.0000	0.0000	0.0000	0.0000	0.0000	0.0000	0.0000
Bryobacteraceae	0.0000	0.0000	0.0000	0.0000	0.0000	0.0000	0.0000	0.0000	0.0000	0.0000
Burkholderiales_unclassified	0.0000	0.0000	0.0000	0.0000	0.0000	0.0000	0.0000	0.0000	0.0000	0.0000
Caldilineaceae	0.0000	0.0000	0.0000	0.0000	0.0000	0.0000	0.0000	0.0000	0.0000	0.0000
Candidatus_Levybacteria_fa	0.0000	0.0000	0.0000	0.0000	0.0000	0.0000	0.0000	0.0000	0.0000	0.0000
Candidatus_Peribacteria_fa	0.0000	0.0000	0.0000	0.0000	0.0000	0.0000	0.0000	0.0000	0.0000	0.0000
Cellvibrionaceae	0.0000	0.0000	0.0000	0.0000	0.0000	0.0000	0.0000	0.0000	0.0000	0.0000
Chromatiaceae	0.0000	0.0000	0.0000	0.0000	0.0000	0.0000	0.0000	0.0000	0.0000	0.0000
Chthoniobacterales_unclassified	0.0000	0.0000	0.0000	0.0000	0.0000	0.0000	0.0000	0.0000	0.0000	0.0000
Chthonomonadaceae	0.0000	0.0000	0.0000	0.0000	0.0000	0.0000	0.0000	0.0000	0.0000	0.0000
Clade_I	0.0000	0.0000	0.0000	0.0000	0.0000	0.0000	0.0000	0.0000	0.0000	0.0000
Clade_II	0.0000	0.0000	0.0000	0.0000	0.0000	0.0000	0.0000	0.0000	0.0000	0.0000
Colwelliaceae	0.0000	0.0000	0.0000	0.0000	0.0000	0.0000	0.0000	0.0000	0.0000	0.0000
Coriobacteriaceae	0.0000	0.0000	0.0000	0.0000	0.0000	0.0000	0.0000	0.0000	0.0000	0.0000
Corynebacteriales_Incertae_Sedis	0.0000	0.0000	0.0000	0.0000	0.0000	0.0000	0.0000	0.0000	0.0000	0.0000
Coxiellaceae	0.0000	0.0000	0.0000	0.0000	0.0000	0.0000	0.0000	0.0000	0.0000	0.0000
CPR2_fa	0.0000	0.0000	0.0000	0.0000	0.0000	0.0000	0.0000	0.0000	0.0000	0.0000
Cyanobacteriaceae	0.0000	0.0000	0.0000	0.0000	0.0000	0.0000	0.0000	0.0000	0.0000	0.0000
Cytophagaceae	0.0000	0.0000	0.0000	0.0000	0.0000	0.0000	0.0000	0.0000	0.0000	0.0000
Cytophagales_unclassified	0.0000	0.0000	0.0000	0.0000	0.0000	0.0000	0.0000	0.0000	0.0000	0.0000
env.OPS_17	0.0000	0.0000	0.0000	0.0000	0.0000	0.0000	0.0000	0.0000	0.0000	0.0000
Erysipelatoclostridiaceae	0.0000	0.0000	0.0000	0.0000	0.0000	0.0000	0.0000	0.0000	0.0000	0.0000
Family_III	0.0000	0.0000	0.0000	0.0000	0.0000	0.0000	0.0000	0.0000	0.0000	0.0000
Fimbriimonadaceae	0.0000	0.0000	0.0000	0.0000	0.0000	0.0000	0.0000	0.0000	0.0000	0.0000
Frankiaceae	0.0000	0.0000	0.0000	0.0000	0.0000	0.0000	0.0000	0.0000	0.0000	0.0000
Gaiellales_unclassified	0.0000	0.0000	0.0000	0.0000	0.0000	0.0000	0.0000	0.0000	0.0000	0.0000
Gemellaceae	0.0000	0.0000	0.0000	0.0000	0.0000	0.0000	0.0000	0.0000	0.0000	0.0000
Geminicoccaceae	0.0000	0.0000	0.0000	0.0000	0.0000	0.0000	0.0000	0.0000	0.0000	0.0000
Gemmataceae	0.0000	0.0000	0.0000	0.0000	0.0000	0.0000	0.0000	0.0000	0.0000	0.0000
Gimesiaceae	0.0000	0.0000	0.0000	0.0000	0.0000	0.0000	0.0000	0.0000	0.0000	0.0000
Hyphomicrobiaceae	0.0000	0.0000	0.0000	0.0000	0.0000	0.0000	0.0000	0.0000	0.0000	0.0000
Iamiaceae	0.0000	0.0000	0.0000	0.0000	0.0000	0.0000	0.0000	0.0000	0.0000	0.0000
IMCC26256_fa	0.0000	0.0000	0.0000	0.0000	0.0000	0.0000	0.0000	0.0000	0.0000	0.0000
Isosphaeraceae	0.0000	0.0000	0.0000	0.0000	0.0000	0.0000	0.0000	0.0000	0.0000	0.0000
JG30-KF-CM45	0.0000	0.0000	0.0000	0.0000	0.0000	0.0000	0.0000	0.0000	0.0000	0.0000
Kaistiaceae	0.0000	0.0000	0.0000	0.0000	0.0000	0.0000	0.0000	0.0000	0.0000	0.0000
KD4-96_fa	0.0000	0.0000	0.0000	0.0000	0.0000	0.0000	0.0000	0.0000	0.0000	0.0000
Legionellaceae	0.0000	0.0000	0.0000	0.0000	0.0000	0.0000	0.0000	0.0000	0.0000	0.0000
Limnochordia_fa	0.0000	0.0000	0.0000	0.0000	0.0000	0.0000	0.0000	0.0000	0.0000	0.0000
LWQ8	0.0000	0.0000	0.0000	0.0000	0.0000	0.0000	0.0000	0.0000	0.0000	0.0000
Magnetospiraceae	0.0000	0.0000	0.0000	0.0000	0.0000	0.0000	0.0000	0.0000	0.0000	0.0000
Marinomonadaceae	0.0000	0.0000	0.0000	0.0000	0.0000	0.0000	0.0000	0.0000	0.0000	0.0000
MBA03_fa	0.0000	0.0000	0.0000	0.0000	0.0000	0.0000	0.0000	0.0000	0.0000	0.0000
MB-A2-108_fa	0.0000	0.0000	0.0000	0.0000	0.0000	0.0000	0.0000	0.0000	0.0000	0.0000
Methyloligellaceae	0.0000	0.0000	0.0000	0.0000	0.0000	0.0000	0.0000	0.0000	0.0000	0.0000
Methylophilaceae	0.0000	0.0000	0.0000	0.0000	0.0000	0.0000	0.0000	0.0000	0.0000	0.0000
Microtrichaceae	0.0000	0.0000	0.0000	0.0000	0.0000	0.0000	0.0000	0.0000	0.0000	0.0000
Microtrichales_unclassified	0.0000	0.0000	0.0000	0.0000	0.0000	0.0000	0.0000	0.0000	0.0000	0.0000
Moritellaceae	0.0000	0.0000	0.0000	0.0000	0.0000	0.0000	0.0000	0.0000	0.0000	0.0000

Myxococcaceae	0.0000	0.0000	0.0000	0.0000	0.0000	0.0000	0.0000	0.0000	0.0000	0.0000
Nakamurellaceae	0.0000	0.0000	0.0000	0.0000	0.0000	0.0000	0.0000	0.0000	0.0000	0.0000
Nitrosomonadaceae	0.0000	0.0000	0.0000	0.0000	0.0000	0.0000	0.0000	0.0000	0.0000	0.0000
Oligoflexales_fa	0.0000	0.0000	0.0000	0.0000	0.0000	0.0000	0.0000	0.0000	0.0000	0.0000
Paracaedibacteraceae	0.0000	0.0000	0.0000	0.0000	0.0000	0.0000	0.0000	0.0000	0.0000	0.0000
PeM15_fa	0.0000	0.0000	0.0000	0.0000	0.0000	0.0000	0.0000	0.0000	0.0000	0.0000
Peptostreptococcaceae	0.0000	0.0000	0.0000	0.0000	0.0000	0.0000	0.0000	0.0000	0.0000	0.0000
Polyangiaceae	0.0000	0.0000	0.0000	0.0000	0.0000	0.0000	0.0000	0.0000	0.0000	0.0000
Pseudoalteromonadaceae	0.0000	0.0000	0.0000	0.0000	0.0000	0.0000	0.0000	0.0000	0.0000	0.0000
Reyranellaceae	0.0000	0.0000	0.0000	0.0000	0.0000	0.0000	0.0000	0.0000	0.0000	0.0000
RF39_fa	0.0000	0.0000	0.0000	0.0000	0.0000	0.0000	0.0000	0.0000	0.0000	0.0000
Rhizobiales_unclassified	0.0000	0.0000	0.0000	0.0000	0.0000	0.0000	0.0000	0.0000	0.0000	0.0000
Rhodanobacteraceae	0.0000	0.0000	0.0000	0.0000	0.0000	0.0000	0.0000	0.0000	0.0000	0.0000
Rickettsiaceae	0.0000	0.0000	0.0000	0.0000	0.0000	0.0000	0.0000	0.0000	0.0000	0.0000
Saccharimonadales_fa	0.0000	0.0000	0.0000	0.0000	0.0000	0.0000	0.0000	0.0000	0.0000	0.0000
Saccharospirillaceae	0.0000	0.0000	0.0000	0.0000	0.0000	0.0000	0.0000	0.0000	0.0000	0.0000
Sandaracinaceae	0.0000	0.0000	0.0000	0.0000	0.0000	0.0000	0.0000	0.0000	0.0000	0.0000
Saprospiraceae	0.0000	0.0000	0.0000	0.0000	0.0000	0.0000	0.0000	0.0000	0.0000	0.0000
SAR202_clade_fa	0.0000	0.0000	0.0000	0.0000	0.0000	0.0000	0.0000	0.0000	0.0000	0.0000
SAR324_clade(Marine_group_B)_fa	0.0000	0.0000	0.0000	0.0000	0.0000	0.0000	0.0000	0.0000	0.0000	0.0000
SAR86_clade_fa	0.0000	0.0000	0.0000	0.0000	0.0000	0.0000	0.0000	0.0000	0.0000	0.0000
Schlesneriaceae	0.0000	0.0000	0.0000	0.0000	0.0000	0.0000	0.0000	0.0000	0.0000	0.0000
SC-I-84	0.0000	0.0000	0.0000	0.0000	0.0000	0.0000	0.0000	0.0000	0.0000	0.0000
Selenomonadaceae	0.0000	0.0000	0.0000	0.0000	0.0000	0.0000	0.0000	0.0000	0.0000	0.0000
Shewanellaceae	0.0000	0.0000	0.0000	0.0000	0.0000	0.0000	0.0000	0.0000	0.0000	0.0000
SM2D12	0.0000	0.0000	0.0000	0.0000	0.0000	0.0000	0.0000	0.0000	0.0000	0.0000
Sulfurovaceae	0.0000	0.0000	0.0000	0.0000	0.0000	0.0000	0.0000	0.0000	0.0000	0.0000
Sutterellaceae	0.0000	0.0000	0.0000	0.0000	0.0000	0.0000	0.0000	0.0000	0.0000	0.0000
Tepidisphaeraceae	0.0000	0.0000	0.0000	0.0000	0.0000	0.0000	0.0000	0.0000	0.0000	0.0000
Thermicanaceae	0.0000	0.0000	0.0000	0.0000	0.0000	0.0000	0.0000	0.0000	0.0000	0.0000
Thioglobaceae	0.0000	0.0000	0.0000	0.0000	0.0000	0.0000	0.0000	0.0000	0.0000	0.0000
Thiotrichaceae	0.0000	0.0000	0.0000	0.0000	0.0000	0.0000	0.0000	0.0000	0.0000	0.0000
uncultured	0.0000	0.0000	0.0000	0.0000	0.0000	0.0000	0.0000	0.0000	0.0000	0.0000
uncultured	0.0000	0.0000	0.0000	0.0000	0.0000	0.0000	0.0000	0.0000	0.0000	0.0000
uncultured	0.0000	0.0000	0.0000	0.0000	0.0000	0.0000	0.0000	0.0000	0.0000	0.0000
uncultured	0.0000	0.0000	0.0000	0.0000	0.0000	0.0000	0.0000	0.0000	0.0000	0.0000
Unknown_Family	0.0000	0.0000	0.0000	0.0000	0.0000	0.0000	0.0000	0.0000	0.0000	0.0000
Verrucomicrobiaceae	0.0000	0.0000	0.0000	0.0000	0.0000	0.0000	0.0000	0.0000	0.0000	0.0000
Vibrionaceae	0.0000	0.0000	0.0000	0.0000	0.0000	0.0000	0.0000	0.0000	0.0000	0.0000
WWH38	0.0000	0.0000	0.0000	0.0000	0.0000	0.0000	0.0000	0.0000	0.0000	0.0000
Xenococcaceae	0.0000	0.0000	0.0000	0.0000	0.0000	0.0000	0.0000	0.0000	0.0000	0.0000
