# Supplementary material for: Timing matters: age-dependent impacts of the social environment and host selection on the avian gut microbiota
Source: Microbiome. 2022 Nov 26;10:202. doi: 10.1186/s40168-022-01401-0 (PMC9700942; doi:10.1186/s40168-022-01401-0)
Supplement: Supplementary file 9 — Additional file 8. LMM investigatigating alpha diversity in different sample types across time. [file 40168_2022_1401_MOESM8_ESM.pdf]

Additional file 8. LMM investigatigating alpha diversity in different sample types across time.

Shannon's Diversity Index

**LMM**	**Est**	**SE**	**CI**		**p**
Intercept	0.92	0.11	0.7	1.13	<0.001
**Random Effects**					
δ2	0.17				
τ00 (Rearing nest)	0.01				
ICC	0.07				
N (Rearing nest)	23				
Observations	367				
Marginal R2	0.142				
Conditional R2	0.202				

Significant Pairwise Comparisons

**Sampling Time**	**Groups**	**Est**	**SE**	**t value**	**p**
5-dph	BF adults vs ZF juveniles	-0.5	0.1	-3.5	0.005
35-dph	BF adults vs ZF juveniles reared by BF	-0.6	0.2	-3.4	0.006
	ZF adults vs Heterospecific foster juveniles	-0.6	0.2	-3.4	0.008
100-dph	ZF adults vs ZF juveniles	-0.3	0.1	-2.8	0.048

Faith's Phylogenetic Diversity Index

**LMM**	**Est**	**SE**	**CI**		**p**
Intercept	3.9	0.11	2.88	3.3	<0.001
**Random Effects**					
δ2	0.16				
τ00 (Rearing nest)	0.01				
ICC	0.08				
N (Rearing nest)	23				
Observations	367				
Marginal R2	0.108				
Conditional R2	0.177				

Significant Pairwise Comparisons

**Sampling Time**	**Groups**	**Est**	**SE**	**t value**	**p**
10-dph	BF adults vs ZF adults	-0.4	0.1	-2.9	0.0298
