# Supplementary material for: Timing matters: age-dependent impacts of the social environment and host selection on the avian gut microbiota
Source: Microbiome. 2022 Nov 26;10:202. doi: 10.1186/s40168-022-01401-0 (PMC9700942; doi:10.1186/s40168-022-01401-0)

Additional file 9. Alpha diversity of different sample types based on **(A)** Shannon's diversity index, and **(B)** Faith's phylogenetic diversity at 5, 10 and 35 dph. The lines within the box plots indicate the medians, and the lower and upper boundaries of the boxes indicate the 25th and 75th percentiles, respectively. Whiskers above and below the boxes correspond to 1.5 times the interquartile range (IQR) above and below the 25th and 75th percentiles, respectively.

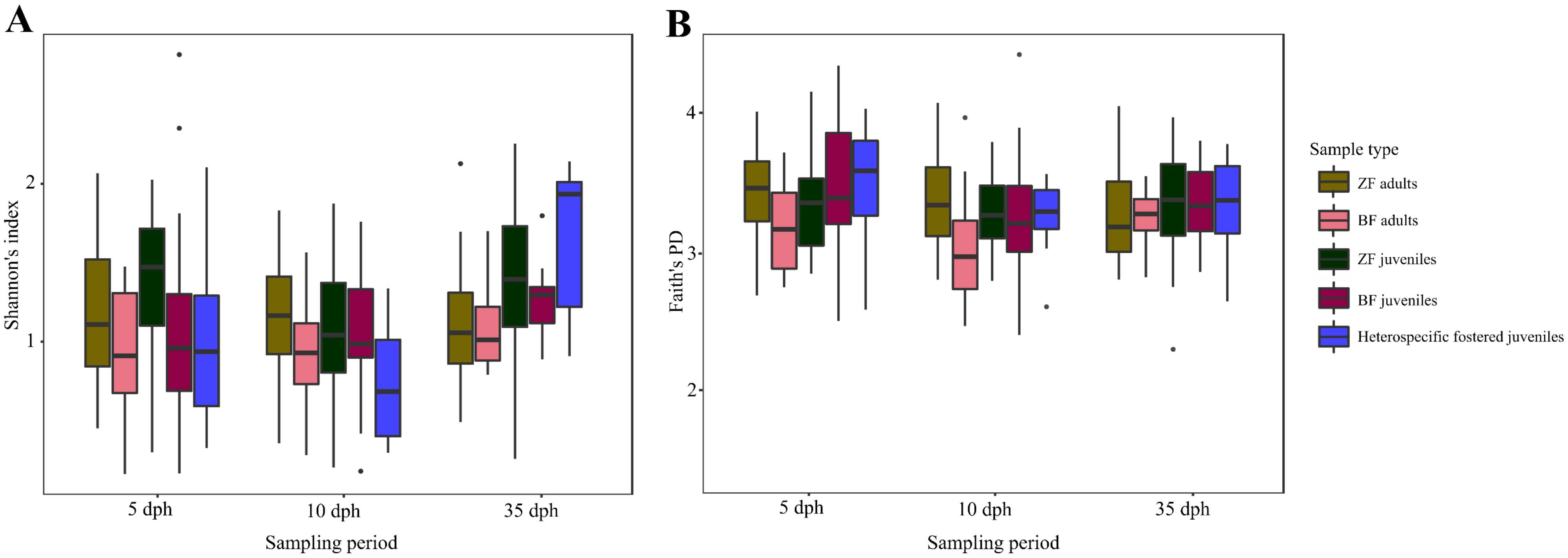

Supplement: Supplementary file 10 — Additional file 9. Alpha diversity of different sample types based on (A) Shannon's diversity index, and (B) Faith's phylogenetic diversity at 5, 10 and 35 dph. The lines within the box plots indicate the medians, and the lower and upper boundaries of the boxes indicate the 25th and 75th percentiles, respectively. Whiskers above and below the boxes correspond to 1.5 times the interquartile range (IQR) above and below the 25th and 75th percentiles, respectively. [file 40168_2022_1401_MOESM9_ESM.pdf]
