# Supplementary material for: Timing matters: age-dependent impacts of the social environment and host selection on the avian gut microbiota
Source: Microbiome. 2022 Nov 26;10:202. doi: 10.1186/s40168-022-01401-0 (PMC9700942; doi:10.1186/s40168-022-01401-0)
Supplement: Supplementary file 11 — Additional file 10. Indicator OTUs for sample types at each sampling time. IndVal index is calculated as the product of specificity (A) and fidelity (B) values ranging between 0 and 1 (1 indicates the taxon exclusively occurs in the given group). [file 40168_2022_1401_MOESM10_ESM.pdf]

Additional file 10. Indicator OTUs for sample types at each sampling time. IndVal index is calculated as the product of specificity (A) and fidelity (B) values ranging between 0 and 1 (1 indicates the taxon exclusively occurs in the given group).

Day	Sample Type	A	B	p.adj	Phylum	Class	Order	Family	Genus
**5-dph**	Heterospecific foster juveniles	0.37	1.00	0.024	*Firmicutes*	*Bacilli*	*Lactobacillales*	*Streptococcaceae*	*Streptococcus*
	Heterospecific foster juveniles	0.96	0.38	0.024	*Proteobacteria*	*Gammaproteobacteria*	*Pasteurellales*	*Pasteurellaceae*	*Unclassified*
	Heterospecific foster juveniles	0.48	0.75	0.016	*Firmicutes*	*Bacilli*	*Lactobacillales*	*Streptococcaceae*	*Streptococcus*
	Heterospecific foster juveniles	0.25	1.00	0.024	*Actinobacteriota*	*Actinobacteria*	*Corynebacteriales*	*Corynebacteriaceae*	*Corynebacterium*
	Heterospecific foster juveniles	0.31	0.75	0.023	*Acidobacteriota*	*Blastocatellia*	*Blastocatellales*	*Blastocatellaceae*	*Unclassified*
**10-dph**	ZF juvenile	0.36	0.93	0.023	*Actinobacteriota*	*Actinobacteria*	*Corynebacteriales*	*unclassified*	*Unclassified*
**35-dph**	Heterospecific foster juveniles	0.54	0.88	0.016	*Firmicutes*	*Bacilli*	*Paenibacillales*	*Paenibacillaceae*	*Paenibacillus*
	Heterospecific foster juveniles	0.49	0.75	0.016	*Firmicutes*	*Bacilli*	*Paenibacillales*	*Paenibacillaceae*	*Paenibacillus*
**100-dph**	BF Adults	0.69	0.82	0.016	*Campilobacterota*	*Campylobacteria*	*Campylobacterales*	*Helicobacteraceae*	*Helicobacter*
	BF Adults	0.65	0.82	0.016	*Spirochaetota*	*Brachyspirae*	*Brachyspirales*	*Brachyspiraceae*	*Brachyspira*
